# PD-1/PD-L1 Blockade: Have We Found the Key to Unleash the Antitumor Immune Response?

**DOI:** 10.3389/fimmu.2017.01597

**Published:** 2017-12-04

**Authors:** Zijun Y. Xu-Monette, Mingzhi Zhang, Jianyong Li, Ken H. Young

**Affiliations:** ^1^Department of Hematopathology, The University of Texas MD Anderson Cancer Center, Houston, TX, United States; ^2^Department of Oncology, The First Affiliated Hospital of Zhengzhou University, Zhengzhou, Henan, China; ^3^Department of Hematology, JiangSu Province Hospital, The First Affiliated Hospital of NanJing Medical University, NanJing, JiangSu Province, China; ^4^Graduate School of Biomedical Science, The University of Texas Health Science Center at Houston, Houston, TX, United States

**Keywords:** PD-1, PD-L1, immune checkpoint blockade, biomarker, MSI, TMB, resistance mechanism, combination immunotherapy

## Abstract

PD-1–PD-L1 interaction is known to drive T cell dysfunction, which can be blocked by anti-PD-1/PD-L1 antibodies. However, studies have also shown that the function of the PD-1–PD-L1 axis is affected by the complex immunologic regulation network, and some CD8^+^ T cells can enter an irreversible dysfunctional state that cannot be rescued by PD-1/PD-L1 blockade. In most advanced cancers, except Hodgkin lymphoma (which has high PD-L1/L2 expression) and melanoma (which has high tumor mutational burden), the objective response rate with anti-PD-1/PD-L1 monotherapy is only ~20%, and immune-related toxicities and hyperprogression can occur in a small subset of patients during PD-1/PD-L1 blockade therapy. The lack of efficacy in up to 80% of patients was not necessarily associated with negative PD-1 and PD-L1 expression, suggesting that the roles of PD-1/PD-L1 in immune suppression and the mechanisms of action of antibodies remain to be better defined. In addition, important immune regulatory mechanisms within or outside of the PD-1/PD-L1 network need to be discovered and targeted to increase the response rate and to reduce the toxicities of immune checkpoint blockade therapies. This paper reviews the major functional and clinical studies of PD-1/PD-L1, including those with discrepancies in the pathologic and biomarker role of PD-1 and PD-L1 and the effectiveness of PD-1/PD-L1 blockade. The goal is to improve understanding of the efficacy of PD-1/PD-L1 blockade immunotherapy, as well as enhance the development of therapeutic strategies to overcome the resistance mechanisms and unleash the antitumor immune response to combat cancer.

## Introduction

It is widely known that ligation of programmed cell death protein 1 (PD-1, also known as CD279) (1) with PD-1 ligand 1 (PD-L1, also called B7-H1 or CD274) (2, 3) activates a critical immune checkpoint leading to T cell dysfunction, exhaustion, and tolerance; high-affinity anti-PD-1 or anti-PD-L1 monoclonal antibodies (mAbs) (4), which block PD-1–PD-L1 interaction, can reverse the immune checkpoint, releasing the brake on T cell responses. However, neither PD-1 nor PD-L1 expression is specific for the reversible T cell dysfunction state, and the effect of PD-1/PD-L1 blockade can be context-dependent. In addition, PD-1 signaling and the mechanism of action of anti-PD-1/L1 mAbs are not completely understood.

Despite these discrepancies and unknowns, PD-1/PD-L1 blockade has achieved great clinical success in combating cancers. Durable response could also be achieved in PD-L1^−^ patients (5, 6). Nonetheless, a large proportion of patients, including those with PD-L1^+^/PD-1^+^ expression, do not respond to PD-1/PD-L1 blockade. Some rational combination therapies have shown synergy *in vivo* or in clinical trials (as well as immune-related toxicities, unfortunately). This article summarizes functional and clinical studies of PD-1/PD-L1 and the resistance mechanisms for PD-1/L1 blockade, and discusses several important questions arising from the disparate data, with the goal of increasing understanding of PD-1, PD-L1, and PD-1/PD-L1 blockade.

## PD-1 and PD-1 Expression: Markers of T Cell Exhaustion or Activation

Contrary to the common perception that PD-1 and PD-L1 expression is a marker of T cell dysfunction associated with cancer and chronic viral infection, PD-1 and PD-L1 can also be expressed under normal physiologic conditions. PD-1 is expressed on 40–80% of memory T cells but not on naïve T cells in the peripheral blood of healthy human adults, and PD-1 expression levels do not directly affect the cytokine production function of CD8^+^ T cells (7).

PD-1 expression may indicate T cell activation, because PD-1 is expressed only on activated T cells *in vivo*, and not on resting T cells. *PD-1* (*PDCD1*) mRNA is mainly expressed in the thymus *in vivo*, with additional possible distribution in the spleen and lung (1). PD-1 protein can be detected in normal murine thymus and spleen T cells at low levels (8), but is strongly induced on thymocytes and T cells in the spleen and lymph nodes after stimulation with an anti-CD3 mAb *in vitro* (9) and increased on T cells in the spleen and liver after tumor cell injection *in vivo* (10). PD-1 is also expressed on activated B cells *in vitro* after stimulation with anti-IgM antibodies, but was undetectable on activated macrophages or dendritic cells (9, 11). In human reactive tonsils, PD-1 is expressed primarily on T cells, as well as a small subset of follicular dendritic cells (12).

The association of PD-1 expression with antigen-specific T cells has also been illustrated in cancer patients. PD-1 expression was significantly higher on antigen-specific CD8^+^ T cells than other CD8^+^ T cells in metastatic melanoma lesions in the same patients (13). In a melanoma mouse model, compared with tumor-ignorant bystander CD8^+^ T cells, tumor-specific CD8^+^ T cells infiltrating the same tumor had significantly higher levels of PD-1, LAG-3, CD69 (activation marker), and 4-1BB (costimulatory molecule) expression and gained 1,414 activation-related (but not exhaustion-related) accessible chromatin regions (14). Adoptive T cell therapy with cells expanded from PD-1^+^CD8^+^ tumor-infiltrating lymphocytes (TILs), but not from PD-1^−^ or bulk CD8^+^ TILs, showed tumor-reactivity and therapeutic benefit *in vivo* (15).

On the other hand, PD-1 expression is associated with suboptimal costimulation and T cell dysfunction when antigen is presented on non-activated or non-professional antigen-presenting cells (16, 17), and PD-1 expression is often induced by high antigen concentration and prolonged antigen stimulation (18, 19). PD-1 may not be a good T cell activation marker because PD-1 surface expression is not rapidly induced on stimulated CD4^+^/CD8^+^ T cells. PD-1 expression has been shown to be increased 24–48 h after stimulation *in vivo* (20–22), 5–7 days after antigen experience (17), 3–8 days after adoptive transfer of pre-activated antigen-reactive CD8^+^ T cells (14), and 19 days after immunization *in vivo* (19), although *PDCD1* mRNA expression was shown to be increased at an earlier time point, as was the suppression of T-cell function. An *in vivo* kinetics study of T cell response to hepatitis B virus infection also showed that after intrahepatic antigen recognition, CD8^+^ T cells first showed rapid induction and decline of IFN-γ-producing capacity, followed by delayed T cell expansion and an increase in cytolytic activity, and the functional oscillation coincided with strong PD-1 induction on antigen-specific T cells (23).

Furthermore, in a melanoma model, the “exhausted” (showing reduced cytokine production capability) tumor-reactive CD8^+^ T cells, compared with “non-exhausted” bystander CD8^+^ T cells, had *Pdcd1* upregulation but downregulation of genes involved in CD8^+^ T cell survival and function (*Il7r, Bcl2, Cxcr3, Ifngr1*, and *Ifngr2*) (14). In patients with metastatic melanoma, tumor-infiltrating T cells had high PD-1 expression and decreased functional avidity compared with T cells infiltrating normal tissues, whereas circulating peripheral blood T cells had minimal PD-1 expression comparable with that in healthy donors. Smaller fraction of antigen-specific CD8^+^ T cells in metastatic melanoma lesions produced IFN-γ compared with those circulating in blood, which was inversely correlated with PD-1 expression (13). Similarly, PD-1 expression gradually increased in TILs with tumor growth but not on spleen T cells in a melanoma tumor model; although a higher percentage of TILs produced IFN-γ after stimulation *ex vivo* compared with spleen T cells, the amount of IFN-γ produced by TILs was lower, and smaller percentage of TILs produced TNF-α (19). In a colon cancer model, the cellular expression levels of PD-1 on intratumoral T cells inversely correlated with the function of CD8^+^ T cells (24).

During chronic infection with lymphocytic choriomeningitis virus (LCMV), *PDCD1* mRNA levels were upregulated in “exhausted” CD8^+^ T cells with impaired cytokine production and proliferation, but *PDCD1* was not upregulated in functional LCMV-specific memory CD8^+^ T cells during acute viral infection (25). Paradoxically, PD-1 protein expression was not limited to chronic LCMV infection, and PD-1 protein was also transiently expressed on CD8^+^ T cells in acute viral infection and downregulated along with LCMV clearance, suggesting that PD-1 protein expression is not a specific marker of exhaustion (25). In fact, during acute infection with rapid control of the viral infection, PD-1^lo^ cells mainly produced antiviral cytokines and PD-1^hi^ cells were the main mediators of cytotoxicity activity (26). Similarly, during chronic mycobacterial infection *in vivo*, PD-1^+^ T cells were not functionally exhausted (highly proliferative and could differentiate into cytokine-secreting T cells), and probably critical for antigen-specific T cell responses (27). Moreover, during tumor growth in a mouse model, although increased PD-1 and LAG-3 expression was accompanied by decreased T-cell effector function, enhancing fatty acid catabolism increased PD-1 expression and improved T-cell effector function; conversely, inhibiting fatty acid catabolism decreased PD-1 expression and impaired T-cell function (28).

PD-1^hi^ expression also does not mark T cell exhaustion in patients with autoimmune disease or cancer. In patients with rheumatoid arthritis, PD-1^hi^CXCR5^−^CD4^+^ cells are expanded in pathologically inflamed non-lymphoid tissues and are functionally active (promoting B cell responses) (29). In follicular lymphoma patients, PD-1^+^ T cells include both functionally “exhausted” (unable to produce cytokines) PD-1^lo^ T cells and PD-1^hi^ “non-exhausted” follicular helper T cells (CXCR5^+^BCL6^+^CD4^+^, supporting the growth and survival of B cells, and secreting IL-21 and IL-4) (30). Increased PD-1^+^ cells in tumor biopsies have been associated with either favorable prognosis in patients with follicular lymphoma (31, 32), lung cancer (33), ovarian cancer (34), or poor survival in cancer patients (35, 36). Furthermore, in melanoma patients, PD-1^+^ T cell clones are antigen-specific T cell clonotypes with higher functional avidity and reactivity (IFN-γ and TNF-α production after activation) than PD-1^−^ T cell clones (37), and PD-1 expression can be used as a biomarker for neoantigen-specific T cells in TILs and in the peripheral blood (38–40). The discrepancies in association of PD-1 expression with T-cell function (exhaustion or avidity) may reflect the complex interplay between various driving forces and effectors of the PD-1 pathway, suggesting that factors other than PD-1 are also important for T-cell functionality.

Similar to PD-1, PD-L1 expression can also be a marker of immune activation. PD-L1 is often not expressed in cell lines *in vitro* but is induced on tumors and in the tumor microenvironment (exceptions include some lymphoma and myeloma cell lines) (10, 41). IFN-γ produced by effector T cells soon after but not before activation of immune response (23), is the major inducer of PD-L1 expression at the transcription level (42). Supporting this, in metastatic melanoma samples, PD-L1^+^ cell densities were shown to significantly correlate with CD8^+^ T cell densities in the tumor and at the invasive tumor margin (43). IFN-γ and TLR ligands induce PD-L1 through the JAK/STAT/IRF-1, MEK/ERK, and MyD88/TRAF6 pathways (44–47). JAK2 (46), MEK/ERK, and p38 MAPK (48) signaling pathways were critical for PD-L1 expression in Hodgkin lymphoma cells. Furthermore, PD-L1 expression is also induced on immune cells after immune activation, including dendritic cells, macrophages, B cells (8, 11), T cells (49), and natural killer cells (50), and this is mediated through the cytokine/chemokine and STAT3 pathways (50–52).

Immune responses are not the only processes that can induce PD-L1 expression; tumor-intrinsic oncogenic pathways can also upregulate PD-L1 expression. For example, oncogenic c-Jun (AP-1) and STAT3 signaling (53), and hypoxia-inducible factor HIF-1α (54) upregulate PD-L1 expression transcriptionally; the oncogenic epigenetic writer EZH2 (55) and epigenetic reader BET4 upregulate PD-L1 (56), whereas the epigenetic eraser histone deacetylase downregulates PD-L1 expression (57). In addition, loss of PTEN function and oncogenic activation of the PI3K/AKT/mTOR pathway increase PD-L1 expression posttranscriptionally (58, 59) [however, *in vivo* PTEN loss did not always affect PD-L1 expression significantly (60)]. Moreover, CSN5, induced by NF-κB p65 (61), and novel CMTM6/4 transmembrane proteins (62, 63) decrease ubiquitination and stabilize PD-L1. EGF signaling induces PD-L1 glycosylation and antagonizes GSK3β-mediated PD-L1 phosphorylation and degradation (64). Enhanced glycolysis and lactate production activate transcriptional coactivator TAZ and induce PD-L1 expression on tumor cells (65). The glycolytic intermediate pyruvate can also metabolically control PD-L1 expression on macrophages through the BMP4/p-SMAD1/5/IRF-1 signaling pathway (66).

Furthermore, PD-L1 is also expressed under normal conditions in both lymphoid and non-lymphoid tissues on human placental trophoblasts, myocardial endothelia cells, and cortical thymic epithelial cells (8, 11, 42), which is involved in peripheral tolerance and immune privilege (67–69). PD-L1 expression has been correlated with either poorer or better survival of cancer patients (70, 71). Taking together, these findings show that, similar to PD-1, PD-L1 expression is not a specific marker for T cell activation or exhaustion.

## PD-1 and PD-L1 Expression as Driver or Biomarker of Immune Suppression: Tumor-Driven or Host-Driven Evolution

As mentioned above, PD-L1 expression can be either immunogenic (tumor-extrinsic, driven by the immune system) (72) or oncogenic (tumor cell-intrinsic, driven by intrinsic mechanisms in cancer cells). It has been controversial whether the immunogenic and oncogenic PD-L1 expression on tumor cells or PD-L1 expression on activated host immune cells is essential for immune evasion. Recently, four studies addressed this question *in vivo* and showed that although all forms of PD-L1 expression contribute to immune suppression in a non-redundant fashion, the relative roles (i.e., predominant or minor) of immunogenic tumor-derived PD-L1 and host-derived PD-L1 expression in suppressing T cell cytotoxicity and infiltration varied depending on the mouse models used, which had different levels of tumor immunogenicity (73–76). *PD-L1* gene deletion in highly immunogenic MC38 colorectal adenocarcinoma tumors resulted in loss of protection from T cell cytotoxicity, whereas the growth of MC38 tumors in PD-L1/PD-L2-knockout (PD-L1^−/−^/L2^−/−^) mice was as robust as in wild-type mice, which elegantly demonstrated that induced tumor PD-L1 expression directly and sufficiently inhibits antitumor immunity, serving as far more than a marker of an ineffective immune response (74).

Similarly designed experiments demonstrated that oncogenic PD-L1 expression in BRAF.PTEN melanoma tumors only slightly inhibited antitumor immunity (74), whereas immunogenic PD-L1 expression on non-tumor cells was critical for immune evasion. Similarly, in a mouse model of melanoma tumors with low immunogenicity, host PD-L1 and PD-1 expression on non-tumor cells is essential for suppressing antitumor immunity. Therefore, although the prevailing notion is that tumors exploit the PD-1 pathway and evade immune response by actively overexpressing PD-L1, this “adaptive immune resistance mechanism” is largely limited to immunogenic PD-L1 expression (74), which is ultimately driven by the host immune response (72).

Although tumor PD-L1 expression in the MC38 model has a driver role, tumor PD-L1-mediated immune suppression has local limitations, which one study proposed as the “molecular shield” functional model. In this model, PD-L1 forms only a temporal molecular shield to protect PD-L1^+^ tumor cells, and the cytolytic function of T cells against other PD-L1^−^ tumor cells with the same antigen is not impaired (77), likely because a close proximity between PD-1–PD-L1 and immunologic synapses is required for PD-L1 function to disturb the T-cell receptor (TCR)–major histocompatibility complex (MHC) interaction. This functional mode is somewhat like another mechanistic model, in which PD-1–PD-L1 interaction increases T cell motility through inhibition of TCR-driven “stop signals” (78). Consistent with this functional model, two (73, 74) of the four recent studies mentioned above showed that tumor PD-L1 expression can protect only PD-L1^+^ tumor cells from cytolytic T cell killing *in situ*, and not PD-L1^−^ cells *in trans*, conferring a selective growth advantage on PD-L1^+^ tumor cells.

However, as shown in mouse models and in cancer patients, immunogenic tumor PD-L1 expression is heterogeneous (76) and transient (75), which does not support the idea that tumor-derived PD-L1 expression is required for tolerance induction and maintenance or that PD-L1^+^ tumor clones are preferably selected during tumorigenesis. It is postulated that PD-L1^−^ tumor cells escape immune surveillance through alternative mechanisms such as decreased MHC expression, increased PD-L2 expression on PD-L1^−^ tumor cells, stromal remodeling, and epithelial–mesenchymal transition (73), as well as compensatory PD-L1 expression on host cells, including T cells (79–81), antigen-presenting cells, monocytic myeloid-derived suppressor cells (MDSCs), and host tissues (81, 82). The compensatory PD-L1 expression can be both IFN-γ-dependent and IFN-γ-independent (75), and may be able to trigger a vicious cycle of immune suppression in the tumor microenvironment (83). Moreover, PD-1 signaling was recently proposed to affect antigen-presenting cells more than tumor cells owing to the increased CD80/CD86 expression on antigen-presenting cells, given that the CD28 receptor is the primary target for PD-1/SHP2-mediated dephosphorylation, as was newly discovered in that study (84). Therefore, host-derived PD-L1 appears to be indispensable for the inhibitory function of the PD-L1/PD-1 axis. However, whether the minor role of the oncogenic PD-L1 expression in the BRAF.PTEN melanoma model applies to tumor PD-L1 expression upregulated by other tumor-intrinsic mechanisms in different types of cancer is unclear.

Furthermore, the driver role of PD-1 on host T cells in immune suppression is demonstrated by the fact that MC38 tumors were completely cleared in PD-1-knockout (PD-1^−/−^) mice. TILs from PD-1^−/−^ mice had an increased ratio of CD8^+^ cells to regulatory T cells (Tregs) and granzyme expression compared with TILs from wild-type mice. In contrast, MC38 tumors (with immunogenic PD-L1 expression) grew similarly robust in PD-L1^−/−^/L2^−/−^ mice as in wild-type mice; PD-L1^−/−^/L2^−/−^ mice and wild-type mice had similar CD8/Treg ratios and PD-1, granzyme, and Ki-67 expression levels in TILs (74). In addition, earlier studies also showed that blockade of PD-1, but not PD-L1, by genetic deletion or mAbs cleared the tumor growth in tumor models (10, 74, 85), and PD-L1 knockout *in vivo* had no effect on PD-1 expression in TILs (74).

Together, these studies may suggest that immune responses are ultimately regulated by the host rather than the tumor. However, another study showed that continuous antigen encounters and TCR stimulation, rather than factors associated with the tumor microenvironment, induce PD-1 expression and T cell dysfunction (17), which is “imprinted” at the premalignant and early malignant phase and later evolves into a therapeutically irreversible state. In line with the idea of antigen dictation of immune response, increased PD-1 expression in expanded blood CD8^+^ cells from patients following viral immunotherapy was not necessarily a target for improving the efficacy of viral immunotherapy (86); immunogenic personalized mutanome vaccines have induced durable clinical response in melanoma patients (87, 88). However, resistance to personalized neoantigen vaccines can still be developed through β2M deficiency and other unclear mechanisms in some patients in these personal neoantigen vaccine trials, and patients receiving PD-1 blockade combination therapy achieved complete regression (87, 88). Moreover, in a tumor model, although tumor vaccines increased antigen-specific TILs, they did not decrease PD-1 expression, which impaired the effector function of TILs, nor did they decrease the percentage of MDSCs in the tumor lesions (which accumulated since early-stage and accentuated after immunization) (19). In a clinical trial of immunization in patients with metastatic melanoma, the expansion and function (tested *in vivo* and *in vitro*) of stimulated antigen-specific CD8^+^ T cells by cancer vaccines were also regulated by increased PD-1 expression (89).

The critical role of antigen was also shown in a mouse model with LCMV infection: T cells functioned normally during acute (Armstrong strain) infection with transient PD-1 expression but were exhausted during chronic (clone 13) infection with stable PD-1 expression (25). Although exhausted CD8^+^ T cells could be reinvigorated by anti-PD-L1 therapy *in vivo*, T cells became re-exhausted with persistent PD-1 expression if antigen concentration remained high (90). Therefore, persistent tumor antigens appeared to be the dictator for PD-1 expression and T cell re-exhaustion. However, this was not supported by antigen withdrawal *in vivo* experiment. After antigen clearance, exhausted T cells and anti-PD-L1-treated exhausted T cells failed to downregulate PD-1 expression (or T-bet and Eomes expression) and had poor recall response upon antigen re-challenge (90).

A study assessing changes in chromatin accessibility during viral infection revealed that acute LCMV infection resulted in stable (5–10%) and dynamic (≥25%) changes in accessible chromatin regions in antigen-specific effector and memory CD8^+^ T cells. In contrast, chronic infection uniquely enriched accessible chromatin regions for NFAT and Nr4a family transcription factors (including enhancers of the *PDCD1* locus) but partially lost the accessibility to some regions (such as *Satb1* and *Il7r* loci) in exhausted CD8^+^ T cells, although exhausted CD8^+^ T cells and effector CD8^+^ T cells shared chromatin accessibility at promoter regions of key effector-related genes, including *Ifng, Gzma, Gzmk, Fasl*, and *Prf1*, as well at inhibitory receptor genes, including *Tim3, Lag3*, and *Ctla4* (91). Anti-PD-L1 therapy *in vivo* caused only minimal epigenetic profile changes in exhausted T cells; instead, the T cell reinvigoration by PD-L1 blockade resulted from transcriptional rewiring with different transcription factors (NF-κB, Jun:AP-1, IRFs, and CTCF, instead of “partnerless” NFATc1, NFAT:AP-1, Nr4a1, Nur77, Eomes, and Egr2) in the epigenetic landscape (90). The epigenetic inflexibility is thought to contribute to re-exhaustion with antigen stimulation without memory-like recall response after anti-PD-L1 treatment (90), suggesting the importance of host T cell-intrinsic regulatory factors including PD-1.

Similar to this unsustained therapeutic effect in viral infection models, an anti-PD-L1 mAb was shown to have only transient antitumor effects in a mouse model, in contrast to the complete suppression of myeloma growth by gene knockout of PD-1 (85). Anti-PD-L1 therapy *in vivo* led to tumor regression with increased antigen-reactive T cell infiltrate and increased IFN-γ and TNF-α production upon antigen stimulation *ex vivo*. However, PD-L1 blockade had only a moderate effect on gene activation and chromatin accessibility in tumor-infiltrating T cells, including upregulation of a few functionally important genes (including granzyme and serpin genes) and dampened accessibility in limited motifs binding NFAT, NFAT:AP-1, TCF, and bZIP:IRF transcription factors. In contrast, 450 accessible regions (including those accessible for Nr4a and NFAT) were gained in “exhausted” T cells compared with “non-exhausted” T cells before the treatment (14).

Furthermore, in an inducible liver cancer model, dysfunction of antigen-specific T cells lasting for more than 30 days was not rescued either after antigen withdrawal or after a decrease in PD-1 levels in TILs by anti-PD-1/PD-L1 therapy (17), suggesting that the dysfunction state was maintained by multiple factors rather than PD-1 alone. Irreversibility of these TILs, which will be discussed more in later sections, somewhat resembled the unresponsiveness of tolerant/anergic T cells to PD-L1 blockade (92). In these settings, PD-1 appeared to be a biomarker rather than the central driver of immune suppression.

## PD-1 and PD-L1: Functionally Dependent or Independent in Driving Immune Suppression

The receptor and ligand relationship between PD-1 and PD-L1 was discovered by Freeman et al. in 2000 (2), and the relationship between PD-1 and PD-1 ligand 2 (PD-L2, also called B7-DC or CD273) was discovered by Latchman et al. in 2001 (93). PD-1 ligation leads to T cell exhaustion (decreased proliferation and effector function) (25), apoptosis (94, 95), or anergy/tolerance (a hyporesponsive state of T cells to a specific antigen that can be induced by lack of costimulation) (96–99). Functional studies have demonstrated that PD-1 receptor ligation is required for PD-1 to prevent T cell activation, and the inhibitory effect of PD-1 ligation depends on TCR strength (21, 22, 42) and co-localization of PD-1 with CD3 and/or CD28 (20, 100).

Molecularly, PD-1 ligation inhibits CD28-mediated costimulation (2, 20, 93); prevents TCR-driven stop signals (78); inhibits TCR signaling in both CD8^+^ and CD4^+^ T cells; blocks cell cycle progression in ^CD4+^ T cells; downregulates expression of antiapoptotic molecules and proinflammatory cytokines; and upregulates expression of Cbl-b ubiquitin ligase in CD8^+^ T cells (20, 93, 100–104). For B cell-derived PD-1 expression, coligation of the PD-1 cytoplasmic region with the B cell receptor (BCR) inhibited BCR signaling *in vitro* (105). Inhibition of TCR/BCR signaling is mediated by the protein tyrosine phosphatase SHP2, which is recruited to the PD-1 immunoreceptor tyrosine-based switch motif upon PD-1 ligation and dephosphorylates ZAP70 (in T cells), Syk, Igβ, PLCγ2, and ERK (in B/T cells) and other downstream kinases, including PI3K/AKT (20, 93, 102, 105, 106). Although SHP2 can be associated with PD-1 immunoreceptor tyrosine-based switch motif with TCR stimulation in the absence of PD-1 engagement, PD-1 engagement is required to block T cell activation (20).

However, in contrast to these earlier studies, a recent study showed that CD28 and Lck (a kinase associated with CD4/CD8 that phosphorylates CD3/TCR, CD28, and PD-1), but not TCR, were the preferred targets of dephosphorylation by PD-1-bound SHP2 in a biochemical reconstitution system (84). PD-1 co-clustered with CD28 in plasma membrane microclusters in a PD-L1-dependent manner but only partially segregated with TCR in stimulated CD8^+^ T cells. Furthermore, intact cell assays using Jurkat T cells and Raji B cells confirmed that CD28, but not TCR, was dephosphorylated after PD-1 ligation with PD-L1; however, the dephosphorylation was only transient (84).

The downregulated PI3K/AKT pathway in T cells upon PD-1 ligation is important for the cell cycle, proliferation, survival, apoptosis, and metabolism. PD-1 also inhibits the PI3K/AKT pathway by inhibiting phosphorylation of PTEN in the C-terminal tail, which decreases PTEN stability but increases PTEN phosphatase activity (107). Because the PI3K/AKT/mTOR pathway is critical for metabolic reprogramming, PD-1 expression and ligation has been linked to metabolic dysfunction in T cells. As shown *in vitro*, ligation of PD-1 on CD4^+^ T cells inhibited glycolysis (106) and glucose transporter Glut1 as well as transportation and catabolism of glutamine, but augmented lipolysis and fatty acid oxidation (108), which promotes Treg development over that of effector T cells (109, 110). In multiple graft-vs.-host disease (GVHD) models, PD-1 expression was shown to increase levels of reactive oxygen species, which was dependent on oxidative metabolism of fat in both CD4^+^ and CD8^+^ T cells, facilitating CD8^+^ T cell apoptosis (95). Conversely, PD-1/PD-L1 blockade partially decreased the generation of reactive oxygen species and cell death of alloreactive PD-1^hi^, but not PD-1^lo^, T cells and increased the severity of GVHD (95). However, in patients with viral infection, exhausted virus-specific CD8^+^ T cells were dependent on glycolysis with high Glut1 and PD-1 expression and depolarized mitochondria which could be rescued by a signal 3 (111) cytokine IL-12, compared with the non-exhausted CD8^+^ T cells within the same patients with metabolic flexibility of utilizing mitochondrial oxidative phosphorylation to fuel the effector function (112). A recent study showed that *in vivo* hypoglycemia and hypoxia metabolic stress caused CD8^+^ T cell exhaustion (which was independent of the PD-1 pathway however); fatty acid catabolism enhanced in CD8^+^ T cells (which was also observed in melanoma patients) partially preserved antitumor effector functions of CD8^+^ TILs but upregulated (possibly indirectly) PD-1 expression; PD-1 blockade synergizes (but did not change) this metabolic reprogramming in inhibiting tumor growth (28). In a B cell leukemia model with increased PD-1 and PD-L1 expression over time in the leukemic microenvironment, impaired T cell metabolism directly contributed to T cell dysfunction, whereas *in vivo* and *in vitro* PD-1 blockade was not sufficient to improve T-cell function (113).

Opposite to the PD-1 function in suppressing glycolysis, enhanced glycolysis induces PD-L1 expression (65), which in turn promotes glycolysis in tumor cells and restricts T-cell function by metabolically competing for glucose (114). Of note, PD-1 signaling inhibits the PI3K/AKT/mTOR and MAPK/ERK pathways in T cells but PI3K/AKT and MEK/ERK signaling pathways activate PD-L1 expression in tumor cells. Tumor PD-L1 promotes MTORC1 signaling but inhibits MTORC2 and autophagy (115). Metabolic competition or adaptation between tumor cells and T cells (114) may contribute to these contrasting pathways, and the paradoxical results in transplantation models: alloreactive donor T-cells in PD-L1-deficient GVHD mice had increased aerobic glycolysis and oxidative phosphorylation (116), whereas donor PD-L1-deficient T cells in wild-type mice had reduced aerobic glycolysis, oxidative phosphorylation, fatty acid metabolism, and cytokine production (117).

In line with the requirement of PD-1 ligation for its suppressive function, in follicular lymphoma, which has very low PD-L1 expression, only subsets of PD-1^+^ T cells have exhausted phenotypes and function (30, 118). However, exhaustion of terminally differentiated PD-1^hi^CD44^int^CD8^+^ T cells during chronic viral infection appeared not to depend on PD-L1 expression, because anti-PD-L1 mAbs could not rescue these PD-1^hi^ T cells from apoptosis or restore the effector function (119). Moreover, PD-1 and PD-L1 expression may be temporally non-overlapping; a kinetics study observed a rapid but transient burst of IFN-γ production at 4 h after adoptive T cell transfer, whereas loss of IFN-γ expression coincided with delayed strong PD-1 induction (23).

PD-L2, the second PD-1 natural ligand, has higher affinity than PD-L1 for PD-1 (120, 121). However, PD-1–PD-L2 interaction is much less functionally significant than the PD-1–PD-L1 interaction owing to the low expression of PD-L2, and PD-1–PD-L1 interaction is sensitive to PD-L2 competition only when PD-L2 levels are very high (120). In sharp contrast to PD-L1, PD-L2 is rarely expressed in lymphohematopoietic and non-hematopoietic tissues (8, 122), except human placental endothelium and medullary thymic epithelial cells (42). PD-L2 can be induced on dendritic cells, macrophages, activated T cells (8, 11, 21, 42), B cells (123–125), and cancer cells by IL-4 through IL-4R/STAT6 in inflammatory macrophages (126), the NF-κB pathway in dendritic cells (8), and IFN-β/IFN-γ in melanoma cells (47). Furthermore, several studies showed that PD-1 and PD-L1, but not PD-L2, induce T cell tolerance and apoptosis, preventing auto/alloimmune responses (16, 67, 97, 116, 127, 128). These data may suggest that PD-1’s suppressive function is largely dependent on PD-L1 but not PD-L2 expression.

In contrast, PD-L1 and PD-L2 can exert inhibitory function independent of PD-1 by binding to B7-1 (CD80) (129) and RGMb (130), respectively. The binding affinity of PD-L1–CD80 is less than that of PD-1–PD-L1 (49). Studies showed that PD-L1–CD80 interaction, but not PD-L1–PD-1 interaction, is responsible for the induction and maintenance of T cell tolerance (131, 132), and that interaction between PD-L1 and PD-1 does not lead to T cell anergy *in vitro* (77). In contrast, in nonobese diabetic (NOD) mouse models, loss of PD-1, but not PD-L1, on antigen-specific CD4^+^ T cells resulted in increased proliferation of CD4^+^ T cells and infiltration of the pancreas during type 1 diabetes (133).

However, early studies showed that similar to the dependence of PD-1 function on receptor ligation (20), the inhibitory activity of PD-L1 and PD-L2 requires the expression of PD-1 (2, 93); in fact, PD-L1 expression in T cells, natural killer cells, and peripheral tissues can have a costimulatory effect with unknown receptors (3, 50, 117, 134–142). PD-L1 expressed on activated CD8^+^ T cells was shown to promote survival and effector function of CD8^+^ T cells during the contraction phase following immunization/antigen stimulation (134). PD-L1 expression in pancreatic islet beta cells was shown to accelerate allograft rejection, increase CD8^+^ T cell proliferation, and promote autoimmune diabetes (135). Likewise, PD-L1 expression induced on donor T cells augmented GVHD lethality (117). A recent study showed that after CD4^+^ T depletion in hematopoietic cell transplantation *in vivo*, PD-L1–CD80 interaction augmented survival and expansion of donor CD8^+^ T cells, resulting in strong graft-vs.-leukemia effects. In contrast, interaction of PD-L1 in recipient tissues with PD-1 on donor CD8^+^ T cells prevented GVHD (139), suggesting that PD-L1’s inhibitory function depends on PD-1. These contradictory results suggest that PD-L1 interactions with PD-1, CD80, and other unknown receptors have context-dependent functions. Unidentified receptors of PD-L2 with stimulatory function have also been reported (143–145).

## PD-1 Blockade and PD-L1 Blockade by Gene Knockout or Antibodies: Efficacies and Limitations

Blocking of the PD-1/PD-L1 pathway by genetic deletion or using anti-PD-1/PD-L1 antibodies has been studied in various preclinical models and the results are quite variable, likely owing to the different roles of PD-1 and PD-L1 in different genetic and immunologic settings. Unlike CTLA-4 germline knockout CTLA-4^−/−^ mice, which spontaneously and rapidly developed fatal lymphoproliferative disease with massive expansion of activated T cells (146, 147), PD-1^−/−^ mice with different genetic backgrounds slowly developed lupus-like proliferative arthritis, glomerulonephritis, splenomegaly, or dilated cardiomyopathy with high-titer autoantibodies in early PD-1 studies (148–150), suggesting that PD-1 can inhibit B cell proliferation and differentiation. In a later study, PD-1 knockout in NOD mice specifically accelerated the onset and frequency of type I diabetes, with strong T helper 1 (Th1) polarization of T cells infiltrating into islets (151). Loss of PD-1, but not PD-L1, was further confirmed to be responsible for the proliferation and infiltration of reactive CD4^+^ T cells during type 1 diabetes in an adoptive T cell transfer model (133). PD-1 also plays a role in positive and negative selection of T cells, as indicated by the altered thymocyte repertoire in PD-1^−/−^ TCR-transgenic mice (152) and *in vitro* (104).

In contrast, PD-L1^−/−^ mice appeared normal but were susceptible to experimental autoimmune hepatitis (induced by accumulation of antigen-activated CD8^+^ T cells in the liver) (153) and experimental autoimmune encephalomyelitis (induced by myelin-reactive CD4^+^ Th1 cells) (81). PD-L1^−/−^ lupus-susceptible (MRL^+/+^) mice developed autoimmune myocarditis and pneumonitis with increased PD-1^+^ macrophage and T cell infiltrates in the heart and lung (154). PD-L2^−/−^ mice exhibited enhanced antigen-specific T cell response and breakdown of oral tolerance compared with wild-type controls (155).

In tumor-formation models, PD-1^−/−^ mice completely suppressed the tumorigenesis of PD-L1^+^ myeloma cells (85); PD-1 deficiency also inhibited the hematogenous dissemination of poorly immunogenic tumors (which were PD-L1^−^ *in vitro*) in PD-1^−/−^ mice (10, 85). In viral infection models, both PD-L1^−/−^ (25) and PD-1^−/−^ mice (156) died from immunopathologic damage within a week after being infected with the LCMV clone 13 strain, which causes chronic infections in wild-type mice. However, both PD-L1^−/−^ and PD-1^−/−^ mice exhibited normal T cell responses to acute LCMV infection and controlled the infection as the wild-type mice did (25, 156). The lethal consequence of chronic infection was a result of systemic vascular leakage due to severe perforin-mediated cytolysis with enhanced CD8^+^ T cell activity (156). These results may suggest that the effectiveness of antiviral immune response is determined by the strain of virus or antigen but not the PD-1/PD-L1 axis, whereas high cytolytic activity due to PD-1/PD-L1 absence results in immunopathologic tissue damage over a prolonged period (23). These results may also suggest that PD-1/PD-L1 interaction has a positive role in generating effective antiviral responses. Indeed, further studies showed that *PD-1* deletion in virus-specific CD8^+^ T cells enhanced T cell proliferation in the acute phase, but overstimulation and robust proliferation lead to increased apoptosis during the contraction phase, as well as accumulation of more cytotoxic but terminally differentiated (Eomes^hi^ cells evolved from T-bet^hi^ progenitor cells), “deeply exhausted” CD8^+^ T cells during chronic LCMV infection (157).

Interestingly, PD-L1 blockade with anti-PD-L1 antibodies during the early-phase (on days 4–6) of systemic LCMV clone 13 infection also caused vascular permeability and ultimately fatal circulatory collapse (156), but anti-PD-L1 therapy on days 23–40 after infection restored the function of exhausted CD8^+^ T cells (proliferation, cytokine production, degranulation, and viral control) with or without CD4^+^ T cell depletion (25). Although CD4^+^ T cell help is critical for sustained CD8^+^ T cell cytotoxic function during chronic LCMV infection (158), other studies showed that combining PD-L blockade with CD4^+^ T cell depletion (159) or Treg cell depletion (160) could rescue deeply exhausted CD8^+^ T cells during the late stage of infection and may result in a significant reduction in viral load.

Although the autoimmune diseases against self-antigens were much milder and at later onset in PD-1/PD-L1/L2 deficient mice than in CTLA-4^−/−^ mice, anti-PD-1 mAbs exhibited stronger antitumor effects than anti-CTLA-4 mAbs in tumor models (10, 25). The enhanced antitumor immunity is believed to result from the occupancy of the PD-1 receptor by anti-PD-1 mAbs which prevents PD-1 from interacting with its natural ligands PD-L1/L2. It has been demonstrated that PD-1 blockade with anti-PD-1 mAbs can increase proliferation and cytokine production of antigen-specific T cells (4, 10, 161), expand intratumoral frequencies of CD8^+^ effector memory T cells (162), enhance the cytotoxicity activity of effector T cells (preferably PD-1^+^ memory T cells with higher functional avidity) (37), augment recruitment of effector cells into the tumor site (4, 10, 161), decrease T cell mobility and enhance stable T–dendritic cell interaction (78), and promote CD8^+^ T cell priming (97, 163) [however, some studies showed that PD-1 blockade alone did not affect CD8^+^ T cell priming and costimulation from CD27 or CD28 may also be required for T cell priming (164, 165)].

In addition, PD-1 expression was found on 64% of freshly isolated natural killer cells from patients with multiple myeloma (166). Anti-PD-1 treatment *in vitro* with a CT-011 antibody (however, its specificity for PD-1 has been questioned) enhanced natural killer cell trafficking, immune complex formation, and cytotoxicity against PD-L1-bearing multiple myeloma cells (166). In multiple tumor models, IL-18 upregulated PD-1 expression on mature natural killer cells only in lymphoid organs but not in tumors; anti-PD-1 therapy *in vivo* abrogated IL-18-mediated metastases (167). PD-1 expression was also found on tumor-associated macrophages in patients with colorectal cancer (168) and on tumor-infiltrating myeloid dendritic cells in ovarian cancer patients (169). PD-1/PD-L1 blockade alone or combined with anti-CD47 therapy *in vivo* increased macrophage phagocytosis but decreased tumor growth and increased survival of mice (168). PD-1 blockade *in vitro* or *in vivo* enhanced dendritic cell function, including cytokine (TNF-α and IL-6) release, antigen presentation, and costimulation owing to NF-κB activation. PD-L1 blockade also increased cytokine release although to less extent (169, 170).

Similar to PD-1 blockade, PD-L1 blockade with anti-PD-L1 mAbs was also shown to increase cytokine production of T helper cells, enhance the cytolytic activity of cytotoxic T cells, and lengthen the duration of antigen-driven T cell migration arrest *in vitro* (78, 100, 171, 172). PD-L1 blockade strongly enhanced proliferation and cytokine production of memory or recently activated T cells from peripheral blood of healthy donors *ex vivo*, but only slightly enhanced naive T cell activation during a primary response (173). In contrast, a study showed that anti-PD-1/PD-L1 mAbs *in vivo* enhanced IFN-γ production but inhibited naïve CD4^+^ T cell proliferation, mediated by IFN-γ from CD4^+^ T cells and nitric oxide from macrophages (174). In a murine model of chronic colitis induced by adoptive transfer of CD4^+^CD45RB^hi^ T cells, PD-L1 blockade treatment before (but not after) the onset of severe colitis suppressed T cell expansion and Th1 cytokine production and prevented the development of colitis (141).

However, the effects of PD-1/PD-L1 blockade were contextual in viral infection models. PD-L1 blockade and PD-1 blockade were effective only for exhausted T cells during chronic LCMV infection, and they did not increase virus-specific CD8^+^ T cells during acute infection (25, 26). Moreover, in a chronic LCMV infection model, PD-L1 blockade rescued only the rescuable subset of exhausted CD8^+^ T cells and not the more terminally differentiated (PD-1^hi^CD44^int^) subset of CD8^+^ T cells (119). Similarly, adoptive transfer of CXCR5^+^CD44^hi^ but not CXCR5^−^CD44^lo^CD8^+^ T cells (the former had PD-1^lo^TIM-3^lo^ expression and higher effector function) reduced the viral load in mice chronically infected with LCMV; the therapeutic effect was further enhanced with anti-PD-L1 combination (175). T cell terminal differentiation during chronic viral infection was also shown to be associated with the Eomes^hi^PD-1^hi^BLIMP-1^+^T-bet^lo^ phenotype (converted from T-bet^hi^PD-1^int^ cells) and increased cytotoxicity but decreased co-production of IFN-γ and TNF-α (176); the therapeutic reversibility of Eomes^hi^PD-1^hi^T-bet^lo^ cells compared with T-bet^hi^PD-1^int^ cells was not examined in that study (176). Another study demonstrated opposite results, showing that anti-PD-L1 or anti-PD-1 therapy during chronic viral infection *in vivo* expanded only the TCF1^+^ memory-like CD8^+^ T cells with PD-1^hi^T-bet^lo^Eomes^+^ expression but not terminally differentiated TCF1^−^CD8^+^ T cells (177). However, whether the effector functions of expanded TCF1^+^CD8^+^ T cells were restored was not shown.

PD-1/PD-L1 blockade also had no effect on established T cell anergy in autoimmune models (92) nor on “non-reversible” dysfunction of T cells in tumor models. In a breast cancer mouse model, the PD-1^hi^-expressing CD8^+^ T cell population failed to be rescued by anti-PD-1 therapy, showing increases in the Treg/CD8^+^ T ratio, in contrast to CD8^+^ T cells with PD-1^lo^ surface expression, which were sensitive to anti-PD-1 mAb in a colon cancer mouse model (24). Several studies demonstrated that the therapeutic reversibility correlated to the duration of dysfunction. In a tamoxifen-inducible autochthonous liver cancer model, dysfunctional tumor-specific CD8^+^ T cells could be rescued by PD-1/PD-L1 blockade in the early-phase, but after 30 or more days the dysfunction was irreversible (17). Notably, this timing effect is opposite to that for PD-L1 blockade during systemic LCMV infection [fatal during the early-phase (156) but effective on days 23–40 (25)]. Also, PD-1 blockade at early time points following viral immunotherapy did not improve durable control of metastatic disease *in vivo* despite the high frequency of PD-1^+^TIM-3^+^CD8^+^ T cells (86).

Transcriptional factors (17) and epigenetic programs may define the function of tumor-specific T cells in TILs and therapeutic reprogrammability (178). Dysfunctional TILs were found to lose access to some intergenic/intragenic regions (probably enhancers), including those in *Ifng, Cd5*, and *Tcf7*, but gain access to some NFATC1-binding sites, including those in *Pdcd1, Ctla4, Cd38*, and *Egr1/2*. The reprogrammability of dysfunction, as assessed by whether the ability to produce IFN-γ and TNF-α was regained after anti-PD-1/PD-L1 therapy, is associated with the discrete chromatin state of T cells—i.e., the “plastic dysfunctional state” at early tumorigenesis and the “fixed dysfunctional state” after day 14–35—and the differential expression of TCF and NFAT family transcription factors. The chromatin changes associated with the fixed dysfunction state included closed TCF/FOS motifs and opened E2F/ETS/KLF motifs. Antigen exposure in tumors has a pivotal role in determining the chromatin state in T cells, whereas PD-1^hi^-expressing CD8^+^ T cells can be in either a plastic or fixed dysfunctional state (178).

However, PD-L1 and B7/CD28 expression in these viral infection models and tumor models, which may be relevant for the therapeutic efficacy, were unclear. For example, terminal differentiated TILs with reduced IFN-γ production may induce very low PD-L1 expression, contributing to the hyporesponsiveness to anti-PD-1/L1 therapy, if pre-existing PD-1–PD-L1 interaction is required for the anti-PD-1/L1 therapy to have a positive effect. It has been shown *in vitro* that PD-1 engagement with anti-PD-1 mAbs inhibited rather than enhanced CD4^+^ T cell expansion and cytokine production with optimal ICOS or suboptimal CD28 costimulation (20, 21, 101) and inhibited glycolysis and glutamine catabolism in T cells (106, 108). However, it is unclear why anti-PD-1 mAbs do not activate similar inhibitory signaling in T cells after blocking the PD-1–PD-L1 interaction in PD-L1^+^ tumors. Also unknown are whether after anti-PD-1 mAbs occupy PD-1, blocked PD-L1 will bind to the alternative CD80 receptor and whether the PD-L1–CD80 interaction in tumors is inhibitory or stimulatory.

In contrast, anti-PD-L1 mAbs, which do not bind to PD-1, should not induce *de novo* inhibitory signaling in T cells in PD-L1^−^ tumors. In addition, anti-PD-L1 mAbs block both PD-1 and CD80 interaction with PD-L1, suggesting that anti-PD-L1 mAbs may have higher efficacy than anti-PD-1 antibodies in PD-L1^+^ tumors. However, treatment with anti-PD-L1 mAbs will not block PD-1–PD-L2 interaction or decrease PD-1 expression, and PD-L1 is broadly expressed in normal tissues, which may suggest that anti-PD-L1 mAbs are less efficacious in PD-1^+^ PD-L2^+^ scenarios but have more immune-related toxicities than anti-PD-1 mAbs.

In preclinical models, comparison between PD-1 blockade and PD-L1 blockade showed inconsistent or contradictory results. Several studies demonstrated that PD-1 and PD-L1 blockade had similar efficacy in preclinical models with PD-L1^+^ tumors (19, 77). In tumor-formation mouse models, PD-1 blockade showed striking efficacy in inhibiting hematogenous dissemination of tumor cells with poor immunogenicity, but PD-L1 blockade had no effect (10). However, PD-L1 blockade was more effective than PD-1 blockade in restoring the function of exhausted T cells in PD-L1-expressing mice with chronic viral infection (25). Moreover, an antibody against PD-L1 on myeloid dendritic cells improved T cell antitumor immunity, although it did not block PD-1–PD-L1 interaction (179). PD-L1 blockade had a stronger effect than PD-1 blockade in breaking T cell anergy *in vivo* in an OT-1 T-cell anergy model. Anergy prevention required early treatment with PD-1 or PD-L1 antibodies after tolerogen exposure, whereas delayed treatment had no effect in preventing T cell anergy (127). The ineffectiveness of PD-1/PD-L1 antibodies in breaking established T cell tolerance, in sharp contrast to the effectiveness in preventing tolerance induction, was also observed in other mouse models (92, 97, 159). In contrast, an anti-PD-L1 mAb, which specifically blocks PD-L1/CD80 but not PD-L1/PD-1 interaction (131), was able to break the pre-established T-cell anergy. However, another study showed that in NOD mice, both PD-L1 and PD-1 blockade enhanced the interactions of tolerized T cells with antigen-bearing dendritic cells, abrogated tolerance, and induced rapid development of autoimmune diabetes, whereas CTLA-4 blockade or anti-CD80 had no such effects (78).

In addition to anti-PD-1 antibodies, small-molecule compounds and peptide antagonists have been reported to inhibit the interaction between PD-1 and PD-L1 (180–183), but their clinical efficacies and dependence on PD-1/PD-L1 expression are currently unknown.

## Clinical PD-1 Blockade and PD-L1 Blockade in Cancer Patients: Successes and Failures

Immune checkpoint blockade with anti-CTLA-4, anti-PD-1, and anti-PD-L1 antibodies has changed the paradigm of cancer treatment. Compared with the CTLA-4 antibodies, anti-PD-1/L1 antibodies have the advantage of lower toxicities (184–186). Currently, the US Food and Drug Administration (FDA) has approved two anti-PD-1 mAbs (PD-1 blockade), nivolumab (Opdivo; Bristol-Myers Squibb Co.) and pembrolizumab (KEYTRUDA; Merck and Co., Inc.), and three anti-PD-L1 mAbs (PD-L1 blockade), atezolizumab (TECENTRIQ; Genentech Oncology), avelumab (BAVENCIO; EMD Serono, Inc.), and durvalumab (IMFINZI; AstraZeneca UK Limited), for the treatment of cancer. The approvals were based on a high objective response rate (ORR), durability of response, or improved survival rate as demonstrated in successful clinical trials (Tables 1 and 2).

**Table 1:** Brief summary of the results of anti-PD-1 therapy clinical trials leading to US food and drug administration approval.

Antibody; reference	Clinical trial	Efficacy	PD-L1 biomarker
**Melanoma**			

Pembrolizumab; Robert et al. (187)	Phase 1b KEYNOTE-001 trial in 173 patients with advanced melanoma progressed following ipilimumab and if *BRAF*^v600^ mutation positive, a BRAF and/or MEK inhibitor	ORR: 26%; 88% of responses were durable	Pooled analysis (*n* = 451) by Daud et al. (188): membranous PD-L1 expression (22C3 mAb) in tumor and immune cells was scored 0–5; higher scores were associated with better ORRs, PFS, and OS; with a ≥1% cutoff for PD-L1^+^, HR: 0.51 for PFS and 0.50 for OS; ORR: 8–12% in PD-L1^−^ patients (durable response), 22–53% in PD-L1^+^ patients with a PD-L1 score 2–5

Pembrolizumab; Ribas et al. (189)	Phase 2 KEYNOTE-002 trial in 540 patients with unresectable or metastatic melanoma who were refractory to prior ipilimumab and if *BRAF*^v600^ mutation positive, a BRAF inhibitor	For 2–10 mg/kg pembrolizumab vs. chemotherapy, 6-month PFS: 34–38 vs. 16% (HR: 0.57/0.50, *p* < 0.0001); ORR: 21–25 vs. 4%; see final update according to Hamid et al. (190) on the right	For 2–10 mg/kg pembrolizumab vs. chemotherapy, 24-month PFS: 16–22 vs. <1%; 24-month OS: 36–38 vs. 30% (HR: 0.86/0.74, *p* = 0.117/0.011, non-significant); ORR: 22–28 vs. 4%; DOR: 73–74 vs. 13% of responders had no progression; OS was consistent across PD-L1 groups but pembrolizumab is favored over chemotherapy in PD-L1^+^ patients

Pembrolizumab, first- or second-line alone; Robert et al. (191)	Phase 3 KEYNOTE-006 trial in 834 patients with advanced melanoma previously untreated or received no more than one line of prior systemic therapy	6-month PFS: 47.3 or 46.4%; 12-month OS: 74.1 or 68.4%; ORR: 33.7 or 32.9%	PFS and OS were better in PD-L1^+^ patients compared with PD-L1^−^ patients. Pembrolizumab vs. ipilimumab: better PFS in both PD-L1^+^ and PD-L1^−^ groups (HR: 0.53/0.52 and 0.67/0.76), better OS only in PD-L1^+^ patients (HR: 0.55/0.58)

Nivolumab; Weber et al. (192)	Phase 3 CheckMate 037 trial in 405 patients with advanced melanoma who progressed after ipilimumab or ipilimumab and a BRAF inhibitor if *BRAF*^v600^ mutation positive	ORR 31.7 vs. 10.6% for chemotherapy	ORR with nivolumab vs. with chemo: in PD-L1^+^ patients (surface expression, cutoff: ≥5% tumor cells, Dako; prevalence: 49%), 43.6 vs. 9.1%; in PD-L1^−^ patients, 20.3 vs. 13.0%

Nivolumab; first-line alone; Robert et al. (6)	Phase 3 CheckMate 066 trial in 418 previously untreated patients who had metastatic melanoma without a BRAF mutation	Improved ORR and survival rates compared with dacarbazine: ORR: 40 vs. 13.9%; 1-year OS: 72.9 vs. 42.1%; median PFS: 5.1 vs. 2.2 months (all *p* < 0.001)	ORR improvement in PD-L1^+^ (≥5% tumor cells) patients (prevalence: 35.4%): 52.7 vs. 10.8%; in PD-L1^−^ patients: 33.1 vs. 15.7%. OS improvement: HR for death, 0.30 in PD-L1^+^ patients and 0.48 in PD-L1^−^ patients

Nivolumab alone or combined with ipilimumab, first-line; Larkin et al. (193)	Phase 3 CheckMate 067 trial in 945 previously untreated patients with metastatic melanoma	Median PFS: 11.5 months with nivolumab plus ipilimumab vs. 2.9 months with ipilimumab (*p* < 0.001), or 6.9 months with nivolumab alone (*p* < 0.001)	With nivolumab alone, in PD-L1^+^ patients, median PFS: 14.0 months, ORR: 57.5%; in PD-L1^−^ patients, median PFS: 5.3 months, ORR: 41.3%. Combination benefit showed in PD-L1^−^ patients: with combination, ORR: 54.8%, median PFS: 11.2 months; with nivolumab alone, ORR: 41.3%, median PFS: 5.3 months; PD-L1^+^ cutoff: ≥5% tumor surface expression, Dako 28-8; PD-L1^+^ prevalence: 23.6%

Combined nivolumab and ipilimumab, first-line; Hodi et al. (194)	Phase 2 CheckMate 069 trial in 142 patients with previously untreated advanced melanoma	For combination vs. ipilimumab alone, ORR: 60 vs. 11%; median PFS: 8.9 vs. 4.7 months; 2-year OS: 63.8 vs. 53.6%	PD-L1 positivity (cutoff: ≥5% tumor cells, Dako 28-8; prevalence: 30%) did not correlate with ORR or PFS

Nivolumab and ipilimumab for adjuvant therapy; Weber et al. (195)	Phase 3 CheckMate 238 trial in 906 patients with resected advanced melanoma	12-month PFS with nivolumab vs. with ipilimumab: 70.5 vs. 60.8% (*p* < 0.001)	12-month PFS in PD-L1^+^ (cutoff: ≥5% tumor cells, Dako 28-8) patients (prevalence: ~34%), 81.9 vs. 73.8%; in PD-L1^−^ patients, 64.3 vs. 53.7%

**NSCLC**			

Nivolumab; Brahmer et al. (5)	Phase 3 CheckMate 017 trial in 272 patients with advanced, refractory squamous NSCLC	For nivolumab vs. docetaxel, ORR: 20 vs. 9% (*p* = 0.008); 1-year OS: 42 vs. 24% (*p* < 0.001); median PFS: 3.5 vs. 2.8 months (*p* < 0.001)	Tumor PD-L1 membranous expression (Dako 28-8) was neither prognostic nor correlated with response; PD-L1^+^ prevalence: 52–54, 36, and 31% using cutoffs of ≥1, ≥5, and ≥10%, respectively

Nivolumab; Borghaei et al. (196)	Phase 3 CheckMate 057 trial in 582 patients with advanced, refractory, or relapsed non-squamous NSCLC	For nivolumab vs. docetaxel, ORR: 19 vs. 12% (*p* = 0.02); median OS: 12.2 vs. 9.4 months (*p* = 0.002); 1-year OS: 51 vs. 39%; 1-year PFS: 19 vs. 8%	Tumor PD-L1 membrane expression (Dako 28-8) correlated with greater efficacy; only in PD-L1^+^ patients, nivolumab was superior; PD-L1^+^ prevalence: 53–55, 38–41, and 35–37% using cutoffs of ≥1%, ≥5%, and ≥10%, respectively

Pembrolizumab; Garon et al. (197)	Phase 1 KEYNOTE-001 trial in 495 patients with advanced NSCLC	ORR: 19.4%; median DOR: 12.5 months; median PFS: 3.7 months; median OS: 12.0 months	In PD-L1^hi^ (≥50% tumor cells with membranous expression; anti-PD-L1 clone 22C3, Merck) patients (prevalence: 23.2%), ORR: 45.2%; median PFS: 6.3 months; median OS: not reached

Pembrolizumab; Herbst et al. (198)	Phase 2/3 KEYNOTE-010 trial in 1,034 patients with previously treated PD-L1^+^ (≥1% tumor) advanced NSCLC	For 2 or 10 mg/kg pembrolizumab vs. docetaxel, median OS: 10.4 (*p* = 0.0008) or 12.7 (*p* < 0.0001) vs. 8.5 months; no difference in PFS	In PD-L1^hi^ (≥50%, Dako 22C3) patients (prevalence: 40–44%), median OS: 14.9 months (*p* = 0.0002) or 17.3 (*p* < 0.0001) vs. 8.2 months; median PFS: 5.0 (*p* = 0.0001) or 5.2 (*p* < 0.0001) vs. 4.1 months

Pembrolizumab, first-line alone; Reck et al. (199)	Phase 3 KEYNOTE-024 in 305 patients with PD-L1^hi^ (≥50%) advanced NSCLC	For pembrolizumab vs. chemotherapy, ORR: 44.8 vs. 27.8%; median PFS: 10.3 vs. 6.0 months (*p* < 0.001); 6-month OS: 80.2 vs. 72.4% (*p* = 0.005)	PD-L1^hi^ (≥50%; Dako PD-L1 IHC 22C3 pharmDx assay) prevalence: 30.2%

Pembrolizumab, first-line combination; Langer et al. (200)	Phase 2 KEYNOTE-021 trial in 123 patients with previously untreated advanced, non-squamous NSCLC	For pembrolizumab plus chemo vs. chemotherapy alone, ORR: 55 vs. 29% (*p* = 0.0032); improved PFS (HR: 0.53, *p* = 0.01) no OS improvement; median DOR: 8 vs. 4.9 months	Combination benefit was shown in PD-L1^hi^ (≥50%; prevalence: 27–33%) and PD-L1^−^ (<1%; prevalence: 35–37%) groups but not in the PD-L1^inter^ (1–49%; prevalence: 32–37%) group. ORR: 80, 57, and 26%, respectively; membranous PD-L1 expression, Dako IHC 22C3 pharmDx assay

**Renal cell carcinoma**			

Nivolumab; Motzer et al. (201)	Phase 3 CheckMate 025 trial in 821 patients with advanced clear cell renal cell carcinoma	For nivolumab vs. everolimus, ORR: 25 vs. 5% (*p* < 0.001); median OS: 25.0 vs. 19.6 months (*p* = 0.002); no PFS improvement	Median OS with nivolumab vs. with everolimus: in PD-L1^+^ patients, 21.8 vs. 18.8 months; in PD-L1^−^ patients, 27.4 vs. 21.2 months; PD-L1^+^ cutoff: ≥1% tumor cells, membranous expression, Dako assay; prevalence: 24%

**Classical Hodgkin lymphoma**			

Nivolumab; Younes et al. (202)	Phase 2 CheckMate 205 trial in 80 patients with classical Hodgkin lymphoma that failed to respond to autologous hematopoietic stem cell transplantation and brentuximab vedotin	ORR: 66.3%; 6-month PFS: 76.9%; 6-month OS: 98.7%	High and low tumor PD-L1 H score (prevalence: both 26%) showed correlation with complete response and progression, respectively; H score was calculated by multiplying the% of PD-L1^+^ malignant cells [by double staining with anti-PD-L1 (405.9A11) and anti-PAX5 mAbs] by the average intensity of positive staining (1, 2, or 3+)

Pembrolizumab; Chen et al. (203)	Phase 2 KEYNOTE-087 trial in 210 patients with classical Hodgkin lymphoma that progressed after autologous hematopoietic stem cell transplantation and/or brentuximab vedotin	ORR: 69%; 6-month PFS: 72.4%; 6-month OS: 99.5%; 75.6% of patients had a response for ≥6 months	Clinical activity was seen across all PD-L1 groups defined by PD-L1 intensity score, tumor-membrane staining score, and histiocyte score (QualTek IHC assay); 90.4% of patients had an intensity score of 3; 88.1% had 100% PD-L1^+^ membrane staining; 71.8% had a histiocyte score of 3

**HNSCC**			

Pembrolizumab; Larkins et al. (204)	Phase 1b KEYNOTE-012 trial in 174 patients with recurrent or metastatic HNSCC	ORR: 16%; DOR: 2.4+ to 27.7+ months; 82% had response durations of ≥6 months	PD-L1^+^ (cutoff: ≥1% tumor cells, membranous expression) prevalence: 65%

Nivolumab; Ferris et al. (205)	Phase 3 CheckMate 141 in 361 patients with recurrent HNSCC	For nivolumab vs. standard therapy, ORR: 13.3 vs. 5.8%; median OS: 7.5 vs. 5.1 months (HR: 0.70, *p* = 0.01); 1-year OS: 36.0 vs. 16.6%; no PFS improvement	Nivolumab vs. standard therapy: in PD-L1^+^ patients, median OS: 8.7 vs. 4.6 months, HR: 0.55; in PD-L1^−^ patients, median OS: 5.7 vs. 5.8 months, HR: 0.89; PD-L1^+^ (cutoff: ≥1% tumor cells, membranous expression, Dako 28-8) prevalence: 57.3%

**Urothelial carcinoma**			

Nivolumab; Sharma et al. (206)	Phase 2 CheckMate 275 trial in 270 patients with metastatic urothelial carcinoma	ORR: 19.6%; median OS: 11.30 months for PD-L1^+^ patients, 5.95 months for PD-L1^−^ (<1%) patients	ORR: 28.4 or 23.8% in PD-L1^+^ patients using ≥5% or ≥1% PD-L1^+^ cutoff (prevalence: 31 and 46%, respectively); 16.1% in PD-L1^−^ patients; tumor-membrane PD-L1 expression was evaluated by the Dako PD-L1 IHC 28-8 pharmDx kit

Pembrolizumab; Bellmunt et al. (207)	Phase 3 KEYNOTE-045 trial in 542 patients with advanced urothelial cancer	For pembrolizumab vs. chemotherapy, ORR: 21.1 vs. 11.4% (HR: 0.73, *p* = 0.001); median OS: 10.3 vs. 7.4 months (*p* = 0.002); no PFS improvement	Pembrolizumab was more superior to chemotherapy in patients with ≥10% PD-L1 combined positive score (prevalence: 30.3%): median OS, 8.6 vs. 4.2 months (HR: 0.57, *p* = 0.005); PD-L1 combined positive score was the % of PD-L1^+^ tumor and immune cells relative to tumor cells, Dako PD-L1 IHC 22C3 pharmDx assay

**MSI-H/dMMR solid tumors**			

Pembrolizumab; Le et al. (208)	Phase 2 NCT01876511 trial in 41 patients with progressive metastatic carcinoma	For dMMR vs. mismatch-repair-proficient colorectal cancer, ORR: 40 vs. 0%; immune-related PFS: 78 vs. 11%	

Pembrolizumab; Le et al. (209)	Phase 2 NCT01876511 trial in 86 patients with advanced dMMR cancers (12 types)	ORR: 53%; median PFS/OS: not reached	

Nivolumab; Overman et al. (210)	Phase 2 CheckMate 142 trial in 74 patients with recurrent or metastatic dMMR/MSI-H colorectal cancer	ORR: 31.1%; median DOR: not reached; estimated 1-year OS: 86%	

**Hepatocellular carcinoma**			

Nivolumab; El-Khoueiry et al. (211)	Phase 1/2 CheckMate 040 trial in 154 patients with advanced hepatocellular carcinoma	ORR: 14.3%; DOR: 3.2 to 38.2+ months; 91% of responses lasted 6+ months; 55% of responses lasted 12+ months	Responses were observed regardless of PD-L1 levels (tumor-membrane expression, Dako PD-L1 IHC 28-8 pharmDx assay)

**Gastric cancer**			

Pembrolizumab; Ref^a^ below	Phase 2 KEYNOTE-059 trial in 259 patients with recurrent locally advanced or metastatic gastric or gastroesophageal junction adenocarcinoma	In 7 MSI-H patients (prevalence: 3%): ORR: 57%; DOR: 5.3+ to 14.1+ months	In 143 PD-L1^+^ (≥ 1% PD-L1 combined positive score) patients: ORR: 13.3%; DOR: 2.8 to 19.4+ months; 58% of responses lasted 6+ months; 26% of responses lasted 12+ months; PD-L1 combined positive score was the% of PD-L1^+^ tumor and immune cells relative to tumor cells, Dako PD-L1 IHC 22C3 pharmDx kit

*ORR, objective response rate; PFS, progression-free survival (rate); OS, overall survival (rate); DOR, duration of response; HR, hazard ratio; NSCLC, non-small cell lung cancer; HNSCC, head and neck squamous cell carcinoma; MSI-H, microsatellite instability-high; dMMR, mismatch-repair deficient*.

*^a^https://www.fda.gov/Drugs/InformationOnDrugs/ApprovedDrugs/ucm577093.htm*.

**Table 2:** Brief summary of the results of anti-PD-L1 therapy clinical trials leading to US food and drug administration approval.

Antibody	Clinical trial	Efficacy	PD-L1 biomarker	Reference
**Urothelial carcinoma (bladder cancer)**				

Atezolizumab	Phase 2 IMvigor210 trial in 310 patients with previously treated inoperable locally advanced or metastatic urothelial carcinoma	ORR: 15%; 84% of responses were ongoing; ORR in patients with ≥5% PD-L1 immune cells (IC) score vs. in patients with <1% IC score: 27 vs. 8% or 26 vs. 13% (*p* < 0.0001)	Percentage of PD-L1^+^ immune cells in the tumor microenvironment correlated with response; prevalence of ≥5% PD-L1 IC score: 32%; prevalence for <1% IC score: 33%; Ventana SP142 PD-L1 assay	Rosenberg et al. (212)

Atezolizumab, first-line alone	Phase 2 IMvigor210 trial in 119 patients with cisplatin-ineligible locally advanced or metastatic urothelial cancer	ORR: 23%; 70% of responses were ongoing; median PFS: 2.7 months; median OS: 15.9 months	Responses occurred across all PD-L1 subgroups according to the % of PD-L1^+^ immune cells in the tumor microenvironment; prevalence for ≥5% PD-L1 IC score: 27%; Ventana SP142 PD-L1 assay	Balar et al. (213)

Durvalumab	Phase 1/2 trial (NCT01693562) in 191 patients with locally advanced or metastatic urothelial carcinoma	ORR: 17.8%; median PFS: 1.5 months; median OS: 18.2 months; 1-year OS rate: 55%	ORR in patients with high PD-L1 scores (≥25% tumor cells, Ventana SP263 PD-L1 Assay) vs. in patients with low/0 PD-L1 scores: 26.3 vs. 4.1%	Powles et al. (214)

Avelumab	Phase 1b JAVELIN Solid Tumor trial in 242 patients with refractory metastatic urothelial carcinoma	ORR: 13.3–16.1%; median response duration had not been reached		(215)

**NSCLC (lung cancer)**				

Atezolizumab	Phase 3 OAK trial in 850 patients with previously treated NSCLC	For atezolizumab vs. docetaxel, median OS: 13.8 vs. 9.6 months (*p* = 0.0003); ORR: 14 vs. 13%; DOR: 16.3 vs. 6.2 months	In PD-L1^+^ patients (prevalence: 54%), median OS: 15.7 months with atezolizumab vs. 10.3 months with docetaxel (*p* = 0.0102); in PD-L1^−^ patients, median OS: 12.6 vs. 8.9 months; PD-L1^+^ cutoff: ≥1% tumor or immune cells; Ventana SP142 PD-L1 assay	Rittmeyer et al. (216)

Atezolizumab	Phase 2 POLAR trial in 277 patients with previously treated advanced or metastatic NSCLC	For atezolizumab vs. docetaxel, median OS: 12.6 vs. 9.7 months (*p* = 0.04); ORR: 14.6 vs. 14.7%	PD-L1 on both tumor and immune cells were evaluated, Ventana SP142 PD-L1 assay; compared with docetaxel, OS with atezolizumab was improved in patients with ≥1% score (prevalence: 68%) but not in patients with <1% score (HR 0.59 and 1.04; *p* = 0.005 and 0.87, respectively); ORR with atezolizumab was improved in patients with ≥50% scores (prevalence: 16%), 37.5 vs. 13.0%, but decreased in patients with 5–49% scores (prevalence: 37%), 7.7 vs. 15.6%	Fehrenbacher et al. (217)

**Merkel cell carcinoma (skin cancer)**				

Avelumab	Phase 2 JAVELIN Merkel 200 trial in 88 patients with refractory metastatic Merkel cell carcinoma	ORR 31.8%; 82% of responses were ongoing	ORR: 34.5% in PD-L1^+^ patients (prevalence: ~78%); 18.8% in PD-L1^−^ patients; PD-L1^+^ cutoff: ≥1% tumor cells, detected by Merck anti-PD-L1 clone 78-10	Kaufman et al. (218)

*ORR, objective response rate; PFS, progression-free survival; OS, overall survival; DOR, duration of response; HR, hazard ratio; NSCLC, non-small cell lung cancer*.

Anti-PD-1 mAbs as single agents or combined with chemotherapy or ipilimumab (anti-CTLA-4 mAb) have been approved for the treatment of the following cancers as first-line, second-line, third-line, or later-line therapies: melanoma (6, 184, 187–195), non-small cell lung cancer (NSCLC) (5, 196–200, 219), classical Hodgkin lymphoma (202, 203, 220), renal cell carcinoma (201), head and neck squamous cell carcinoma (HNSCC) (204), urothelial carcinoma (205–207), microsatellite instability-high (MSI-H) cancers (including colorectal cancer and other solid cancers) (208–210), hepatocellular carcinoma (211), and gastric or gastroesophageal junction adenocarcinoma [approval to pembrolizumab (Table 1); however, only nivolumab phase 3 results are available (221)]. Anti-PD-L1 mAbs as single agents in first-line, second-line, or salvage therapies have been approved in urothelial carcinomas (212–215, 222), NSCLC (216, 217), and Merkel cell carcinoma (218). Many clinical trials in different cancer types or settings are still ongoing and some have shown good results, such as the phase 3 PACIFIC clinical trial for durvalumab as consolidation therapy in patients with stage III NSCLC (223). The ORRs with PD-1/PD-L1 blockade as monotherapy in relapse/recurrence settings largely differ by disease entities; the ORR is close to 70% in classical Hodgkin lymphoma which frequently has 9p24 copy number alterations (202), ~40% in skin cancers, ~20% in lung cancers, ~25% in renal cancer, 13–23% in bladder cancer, and 13–16% in HNSCC. PD-1 blockade and PD-L1 blockade largely showed similar efficacy, although the ORRs were ~5% higher with PD-1 blockade than with PD-L1 blockade in NSCLC, and results of PD-L1 blockade need to be validated in phase 3 studies.

However, anti-PD-1/PD-L1 therapies did not work in all cancers [e.g., chronic lymphocytic leukemia (224)]. Although most of responses were more durable than traditional therapies, some patients who initially responded to checkpoint blockade experienced relapse [acquired resistance; however, a small subset of relapsed patients could still respond to continuing blockade therapy; the rate was 3.6% in urothelial carcinoma patients treated with atezolizumab (225)]. Moreover, recently five phase 3 studies have failed to meet the endpoints [first-line nivolumab alone or durvalumab plus tremelimumab compared with chemotherapy; nivolumab, pembrolizumab, or atezolizumab as a later-line therapy compared with chemotherapy or standard treatment (226, 227), Table 3], even though blockade has shown clinical activity in phase 1/2 trials (212, 228–231). Two phase 3 clinical trials of pembrolizumab in multiple myeloma have been placed on full clinical hold owing to increased risk of death.

**Table 3:** Examples of anti-PD-1/L1 clinical trials that missed the endpoint or were discontinued owing to increased risk of death.

Regimen	Clinical trial	Efficacy	Toxicities	Reference
**OPDIVO**				

Nivolumab as first-line monotherapy compared with chemotherapy	Phase 3 CheckMate 026 trial in 423 patients with previously untreated stage IV or recurrent NSCLC with PD-L1 scores ≥5%	For nivolumab vs. chemotherapy, median PFS: 4.2 vs. 5.9 months (HR: 1.15; *p* = 0.25; missed the endpoint); median OS: 14.4 vs. 13.2 months (HR: 1.02)		Carbone et al. (226)

Nivolumab compared with investigator’s choice chemotherapy	Phase 3 CheckMate 037 trial in 405 patients with ipilimumab-refractory advanced melanoma	For nivolumab vs. chemotherapy, higher and more durable responses but no survival improvement: median OS: 16 vs. 14 months; median PFS: 3.1 vs. 3.7 months		Larkin et al. (227)

**KEYTRUDA**				

Pomalidomide and low-dose dexamethasone with or without pembrolizumab	Phase 3 KEYNOTE-183 trial in 249 patients with refractory or relapsed multiple myeloma	ORR: 34% in the pembrolizumab arm vs. 40% in the control arm; time-to-progression: 8.1 vs. 8.7 months (HR: 1.14)	At median follow-up of 8.1 months, 29 deaths in the pembrolizumab arm vs. 21 deaths in the control arm (HR: 1.61)	http://www.onclive.com/web-exclusives/fda-discloses-data-on-halted-pembrolizumab-myeloma-trials

Lenalidomide and low-dose dexamethasone with or without pembrolizumab	Phase 3 KEYNOTE-185 trial in 301 patients with newly diagnosed and treatment-naïve multiple myeloma	ORR: 64% in the pembrolizumab arm vs. 62% in the control arm; HR for time-to-progression: 0.55	At a median follow-up of 6.6 months, 19 deaths in the pembrolizumab arm compared to 9 deaths in the control arm (HR: 2.06)	

Pembrolizumab compared with standard treatment	Phase 3 KEYNOTE-040 trial in 495 patients with previously treated recurrent or metastatic HNSCC	Missed the primary endpoint of OS [HR: 0.82 (95% CI: 0.67–1.01); *p* = 0.03 (one-sided)]		Larkins et al. (204) and Ref^a^ below

**TECENTRIQ**				

Atezolizumab compared with chemotherapy	Phase 3 IMvigor211 trial in 931 patients with previously treated locally advanced or metastatic urothelial cancer	Failed to meet the primary endpoint of improving OS		http://www.roche.com/media/store/releases/med-cor-2017-05-10.htm

**IMFINZI**				

First-line durvalumab alone or combined with tremelimumab compared with chemotherapy	Phase 3 MYSTIC trial in previously untreated metastatic NSCLC	Did not improve PFS of patients with PD-L1 scores ≥25% compared with chemotherapy		Peters et al. (231) and Ref^b^ below

*NSCLC, non-small cell lung cancer; PFS, progression-free survival; HR, hazard ratio; OS, overall survival; ORR, objective response rate; HNSCC, head and neck squamous cell carcinoma; CI, confidence interval*.

*^a^http://www.onclive.com/web-exclusives/pembrolizumab-falls-short-in-phase-iii-head-and-neck-cancer-trial*.

*^b^https://www.astrazeneca.com/media-centre/press-releases/2017/astrazeneca-reports-initial-results-from-the-ongoing-mystic-trial-in-stage-iv-lung-cancer-27072017.html*.

In addition, hyperprogression, a new pattern of disease progression after anti-PD-1/PD-L1 monotherapy, that is associated with elderly age and worse overall survival but not specific tumor types, has been identified in ~9% of cancer patients (232, 233). A higher rate of hyperprogression (regional recurrence in most cases without any cases of pseudoprogression), 29%, was retrospectively identified in patients with HNSCC (234). The different rates may result from differences in hyperprogression definition and size of the cohorts, since in the HNSCC cohort, hyperprogression was significantly associated with shorter progression-free survival but not with overall survival. These unexpected clinical observations may reflect our incomplete understanding of the PD-1/PD-L1 pathway and immune regulation mechanisms.

## Molecular Determinants and Predictive Biomarkers for PD-1/PD-L1 Blockade Immunotherapy: PD-L1^+^, Tumor Mutational Load, T Cell Functional State, or Other Host Factors

**Figure 1:**
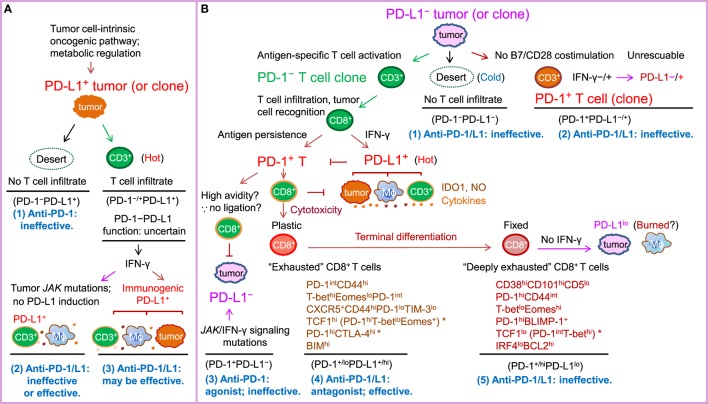
Schematic illustration of PD-1/PD-L1 expression in the tumor setting as a marker of T cell activation and driver of T cell dysfunction, as well as a predictive biomarker for response to PD-1/PD-L1 blockade in PD-L1^−^ and PD-L1^+^ tumors according to the prevailing notion. PD-L2, which is infrequently expressed and potentially has PD-1-independent positive function, is not depicted in the figure for clarity. The PD-L1–CD80 axis is also not illustrated because its role and significance in the cancer setting is unclear. **(A)** In tumors (or tumor clones) with cell-intrinsic PD-L1 expression driven by the oncogenic pathways, whether anti-PD-1/PD-L1 is effective may depend on the activity of the PD-1–PD-L1 axis. If T-cell infiltration is lacking (a “desert”-like immune landscape, or “cold” tumors), or PD-1 is not expressed on T cells, anti-PD-1 therapy will not elicit a *de novo* T cell response. If the tumor is infiltrated with immune cells (“hot” tumor) and the oncogenic or immunogenic PD-L1 expression suppresses T cell activation by binding to PD-1 within the T-cell receptor microclusters, anti-PD-1/PD-L1 therapy can be effective. IDO1, NO (nitric oxide), and suppressive cytokines in the tumor microenvironment may contribute to resistance to PD-1/PD-L1 blockade therapy. **(B)** In tumors without cell-intrinsic PD-L1 expression, tumors (or tumor clones) with low immunogenicity (“cold” tumors) or costimulation may not respond to anti-PD-1/PD-L1 therapy, whereas tumors (or tumor clones) with a high neoantigen load elicit antitumor T cell responses (“hot”) but their response to anti-PD-1/PD-L1 therapy varies. Antigen-specific CD8^+^ T cells secrete IFN-γ, which may turn PD-L1^−^ tumors into PD-L1^+^ tumors infiltrated with PD-L1^+^ macrophages, dendritic cells, and T cells. However, if tumors do not have IFN-γ receptors or have *JAK2* mutations, tumors may remain PD-L1^−^ and not respond to anti-PD-1/PD-L1 treatment or respond if PD-L1 is induced on non-tumor immune cells. In PD-L1^+^ tumors, prolonged antigen stimulation gradually induces PD-1 expression on antigen-specific T cells. PD-1 ligation with PD-L1 induced on tumors, antigen-presenting cells, and T cells in hot tumors in turn suppresses antitumor function of effector T cells, leading to T cell “exhaustion” (a term initially used for T cell dysfunction during chronic viral infection). Early-phase T cell “exhaustion” is plastic and can be reversed by PD-1/PD-L1 blockade; in contrast, if T cell dysfunction is fixed after terminal differentiation, “deeply exhausted” T cells cannot be rescued by PD-1/L1 blockade. Inflexibility in transcriptional and epigenetic programs may contribute to the therapeutic irreversibility of deeply exhausted T cells. Potential markers suggested by studies in tumor models, viral infection models, and cancer patients are summarized below the labels for these two different dysfunctional stages of PD-1^+^CD8^+^ T cells. * indicates disparities in PD-1 levels in the literature (please refer to the text for details).

Given the high cost and potential toxicities of the treatment, efforts have been made to identify predictive biomarkers for selecting patients who are most likely to benefit from anti-PD-1 immunotherapy. PD-L1 is the first and most studied biomarker for PD-1 blockade (188, 235). Theoretically, PD-1 blockade should work only in PD-1^+^ PD-L^+^ patients and not in PD-1^−^ patients (4) or PD-L^−^ patients (most PD-L1^−^ cases are PD-L^−^) (Figure 1), because PD-1 ligation is indispensable for PD-1-mediated suppression, and in the absence of PD-1 natural ligand, anti-PD-1 mAbs can act as PD-1 agonists to inhibit rather than enhance PD-1^+^CD4^+^ T-cell function (20, 21, 101). However, in multiple clinical trials, PD-L1 negativity was not found as an excluding factor for patient selection (Table 1). Durable clinical response to PD-1 blockade was also observed in some PD-L1^−^ patients with unknown PD-L2 status (although with a lower response rate in most studies). Furthermore, in some studies of squamous NSCLC and renal cell carcinoma, the efficacy of PD-1 blockade (response rate or survival outcome) in PD-L1^−^ patients was similar to or even better than that in PD-L1^+^ patients (5, 201). The predictive values of the percentage and cellular levels of PD-1 expression in correlation with PD-L1 expression were unclear in these clinical studies.

Unlike tumor PD-L1 expression, which has shown predictive value for the efficacy of anti-PD-1 therapy in most studies (Table 1), PD-L1 expression on immune cells in the tumor microenvironment was more correlated with treatment response to anti-PD-L1 therapy (212, 236) (Table 2). However, the correlation of PD-L1 expression with response was absent in other studies, with similar ORRs or improved survival rates occurring across all PD-L1 subgroups (213, 216). Even in studies showing correlations, some patients who lacked both tumor and immune cell expression of PD-L1 still responded to anti-PD-L1 therapy (212, 218). The mechanisms of response to anti-PD-1/L1 therapy in these PD-L1^−^ patients are unknown, posing intriguing questions. One plausible explanation would be failure to detect PD-L1 expression owing to technical reasons or temporal and spatial expression (for example, clustered PD-L1 expression in the early time course of T cell activation and the dynamic PD-L1 expression on circulating T cells).

Efforts have been made to improve the prediction accuracy, by standardizing the detection antibodies and immunohistochemistry assays (Tables 1 and 2), separately assessing PD-L1 expression on tumor cells and PD-L1 expression on non-tumor cells, and optimizing PD-L1 cutoffs. Because most studies have used low PD-L1 cutoffs (≥1% or ≥5%), and PD-L1 function is limited to local inhibition, acting as the “molecular shield” of PD-L1^+^ cells, we would postulate that the association of PD-L1 expression with immune activation status, rather than the correlation with immune suppression strength, may underlie the predictive value of PD-L1 expression for anti-PD-L1 therapy. Interestingly, in NSCLC, anti-PD-1 therapy (pembrolizumab) demonstrated superiority over chemotherapy in patients with ≥50% or <1% tumor PD-L1 scores, but this benefit was absent in patients with 1–49% tumor PD-L1 scores (200). This non-linear correlation reappeared in an anti-PD-L1 study in NSCLC, in which atezolizumab compared with docetaxel was associated with improved ORR in the ≥50% PD-L1^+^ group but decreased ORR in the 1–49% group (217). The predictive 50% cutoff of PD-L1 expression has been included in the FDA indication for pembrolizumab in metastatic NSCLC tumors as frontline therapy (199).

A recent biomarker study using longitudinal tumor samples from patients with metastatic melanoma showed that expression of PD-1, LAG-3, and PD-L1 in early on-treatment (median: 1.4 months after initiation of treatment), but not in pre-treatment (median: 3 months prior to treatment), biopsies was highly predictive for response to PD-1 blockade, suggesting the inability to accurately predict the clinical response before anti-PD-1 therapy (237). In this study, some responders had no PD-1/PD-L1 expression in pre-treatment samples but had high immune marker expression in on-treatment samples; conversely, many non-responders had high PD-L1 expression in pre-treatment samples but had low PD-L1 expression in on-treatment samples. The observation that PD-L1^−^ patients turned into PD-L1^+^ patients appeared to suggest that immunogenic PD-L1 expression was induced after anti-PD-1 therapy. However, results from *in vitro* experiments (20, 21, 101) suggest that in PD-L1^−^ patients, binding of anti-PD-1 mAbs to PD-1 will inhibit IFN-γ production, and therefore, the baseline PD-L1^−^ status should not be changed after anti-PD-1 therapy. In contrast to these discrepancies, hyperprogression after anti-PD-1/L1 therapy tended to be associated with PD-L1 negativity (232, 233).

Because PD-L1 expression in on-treatment tumors predicted response to anti-PD-1 treatment (237), one would postulate that inducibility of PD-L1 expression can predict effectiveness of PD-1 blockade. Consistently, JAK2/STAT1 signaling is increased in classical Hodgkin lymphoma (238) which showed high ORRs [(220) and Table 1] to PD-1 blockade. Conversely, *JAK1*/*2* and *APLNR* loss-of-function mutations, which result in non-inducibility of tumor PD-L1 expression by IFN-γ, have been associated with primary or acquired resistance to PD-1 blockade in solid tumors; PD-1 blockade was ineffective for these patients even if their mutational load was high (239–241). However, PD-L1 should still be inducible on nonmalignant immune cells, which did not harbor *JAK1*/*2* and *APLNR* mutations as tumors did, suggesting that other immune escape/suppressive mechanisms may also contribute to the treatment resistance in these patients. Indeed, *JAK1/2* or IFN-γ pathway gene mutations were not always found to be associated with clinical response (242, 243).

Microsatellite instability arising from mismatch-repair deficiency is the second predictive biomarker (208, 244) approved by FDA (245). MSI-H tumors have high levels of neoantigens associated with a strong local and systemic immune response (246). In addition, MSI-H tumors were shown to display upregulation of multiple immune checkpoints, including PD-1, which may limit the vigorous immune microenvironment (247), making PD-1 blockade a rational treatment approach. In metastatic colorectal cancer, the ORR with pembrolizumab was 40% in MSI-H patients compared with 0% in mismatch-repair-proficient patients (208). In an expanded study of advanced mismatch-repair-deficient cancers across 12 different tumor types, the objective radiographic response rate was 53% and the complete response rate was 21% (209).

High tumor mutational burden and neoantigen load, which are fairly common across cancer types compared with the uncommon MSI-H (248), have also been correlated with sensitivity to PD-1 blockade (higher ORR and/or prolonged survival) in melanoma, NSCLC, glioma (243, 249–252), and likely across types of solid cancers (252). In addition, high numbers of indel mutations were found in renal cell carcinomas, and frameshift indel count was associated with response to PD-1 blockade in melanoma patients (253). Conversely, high copy number loss burden was associated with resistance to checkpoint blockade (242). However, classical Hodgkin lymphoma has a high ORR but not a high mutational burden. Some gene mutations may correlate with treatment resistance (such as *JAK2* and *B2M*). Although a study showed that neoantigen load correlated with T-cell infiltration in colorectal cancers (254), another study showed that the density of immunogenic antigens did not correlated with T-cell infiltration and local immunity in melanoma (255). To reduce whole-exome sequencing and enhance the clinical applicability of tumor mutational burden, targeted comprehensive genomic profiling (248) and small next generation sequencing panels (256) have been developed. Progress has been made in understanding the association of response with particular gene (such as DNA repair genes *BRCA2* and *POLE*; potentially also *PMS2, MSH2*/*6*, and *MLH1*) mutations and clonal neoantigens, as well as T cell clones responding to PD-1/L1 blockade (243, 248, 250, 256–259). *POLE* mutations have been shown to be associated with not only higher mutational burden (248) but also immune signatures and lymphocytic infiltration independent of MSI-H status in endometrial cancer (260). However, particular gene mutations and alterations (such as loss of *PTEN* and *CDKN2A*) and mutational burden showed inconsistent significance in studies (60, 242, 243, 258). Tumor mutation load and clonal mutation load (less heterogeneity) were associated with overall survival and response to nivolumab in ipilimumab-naive patients but not in patients who had previously progressed on ipilimumab (243). In the latter group of patients, response to PD-1 blockade was inconsistently associated with T cell clonality (242, 243).

Some T cell-derived biomarkers have also been found to be predictive of response to PD-1 blockade in patients with advanced melanoma; these biomarkers include high baseline CD8^+^ and PD-1^+^ density at the invasive tumor margin and inside the tumor, proximity between PD-1^+^ and PD-L1^+^ cells, clonal TCR repertoire (43, 242), BIM expression in tumor-reactive PD-1^+^CD8^+^ T cells (261, 262), and higher proportion of PD-1^hi^CTLA-4^hi^ cells with a partially exhausted T cell phenotype (capable of producing IFN-γ but lost the ability to produce TNF-α and IL-2) within CD8^+^ TILs (263). Baseline *PDCD1* mRNA expression was also associated with progression-free survival after anti-PD-1 therapy in a pooled cohort of cancer patients (264). However, the findings that PD-1^hi^CTLA-4^hi^ TILs that were preferably expanded after anti-PD-1 therapy in melanoma patients (263) counters the findings in preclinical models [PD-1^hi^ T cells were irreversible (178) and anti-PD-1 therapy was effective only in tumors with low frequencies of PD-1^+^ T cells (24)].

In addition, in preclinical models, low levels of CD38, CD101, and CD30L whereas high levels of CD5 surface expression (178), low to intermediate levels of PD-1 expression on CD8^+^ T cells (24), as well as high TCF1 (177) and IRF4 nuclear expression were associated with T cell plastic dysfunctional state whereas high BCL2 expression in CD8^+^ T cells was associated with fixed dysfunctional state (178). The potential of these biomarkers may be clarified in future anti-PD-1/L1 clinical trials.

Moreover, several non-T host factors, including absolute lymphocyte count, relative eosinophil count, ≤2.5-fold elevation of serum lactate dehydrogenase, and the absence of metastasis other than soft-tissue/lung metastasis, have also been associated with improved overall survival in melanoma patients treated with pembrolizumab (265). However, efficacy comparison with controlled arms (anti-PD-1 therapy compared with traditional therapy) will be more informative (266). Also notably, a retrospective analysis found a five-factor {serum lactate dehydrogenase elevation, age <65 years, female sex, previous ipilimumab treatment [however, this factor was non-significant in the earliest pembrolizumab trial (184)], and liver metastasis} prediction scale was associated with lower ORRs to anti-PD-1 therapy (267). Although studies have shown that response to anti-PD-L1 therapy was associated with a Th1 gene signature in on-treatment samples (236), a recent study found that early decrease of IL-8 (a Th1-associated cytokine) levels in the serum 2-3 weeks after anti-PD-1 therapy was predictive of response in melanoma and NSCLC patients, including rare cases [0.6–4% (268, 269)] with pseudoprogression (270). A prospective trial in melanoma patients found that response to anti-PD-1 therapy induced genomic contraction, which was associated with pronounced pre-existing immune signatures in pre-treatment samples, including TCR/PD-1/IFN-γ/IL-2/PI3K signaling signatures as well as MHC class II and other genes resembling a macrophage signature (243).

The gut microbiome in cancer patients has been shown to influence PD-1 blockade efficacy. Clinical responses to anti-PD-1 immunotherapy were associated with high diversity and relative abundance of Ruminococcaceae bacteria in prospectively collected microbiome samples from patients with metastatic melanoma (271) and relative abundance of *A. muciniphila* in patients with NSCLC, renal cell carcinoma, or urothelial carcinoma (272). In addition, commensal *Bifidobacterium* was shown to confer improved anti-PD-L1 efficacy *in vivo* (273). Mechanisms accounting for the favorable prognosis may include increased tumor infiltration of CD8^+^ T cells, more effector T cells than Tregs in systemic circulation, dendritic cell function, IL-12 secretion, anabolic metabolism, and systemic inflammation (271–273), but the mechanistic links for these immunomodulatory effects remain unknown. PD-1 also regulates the gut microbiota and the function and survival of IgA-producing plasma B cells, but this effect can be abrogated by PD-1 blockade, as was shown *in vivo* (274).

## Overcoming Resistance to PD-1/PD-L1 Blockade: Various Combination Strategies

Like a tug-of-war, the actions of immune response and tumor development resist each other. PD-1 blockade may have antitumor effects in cancer patients (275) but this is not always sufficient for a clinical response. Resistance mechanisms may come from either the immune system or the tumor. The ratio of immunologic reinvigoration to tumor burden, but not the magnitude of reinvigoration alone, was found to be predictive of response to pembrolizumab and overall survival in patients with advanced melanoma (276). Maximized innate and adaptive responses, achieved through combination therapies, were capable to eliminate large, advanced, poorly immunogenic tumors in mice (277).

Multiple tumor- or immune-driven resistance mechanisms have been identified and targeted in combination with PD-1 blockade. First, absence of “signal 1” and T cell activation leads to ineffectiveness of anti-PD-1/L1 monotherapy (278). Studies have shown that *B2M* mutations, deletions, or loss of heterozygosity, which leads to loss of MHC class I expression and failure of antigen recognition, is a potential mechanism for immune escape and resistance to PD-1 blockade in patients with melanoma (239, 279). Clinical outcome of anti-PD-1/PD-L1 therapy was shown to correlate with MHC class II positivity in a unique subset of melanoma cells (typically MHC class II is expressed only on immune cells in solid tumors), as well as increased CD4^+^ and CD8^+^ TILs in melanoma patients (280).

However, a surprisingly high frequencies of decreased or absent expression of β2M/MHC class I (79% overall; 92% in *PD-L1*/*L2* amplified cases) and MHC class II (67%) were found in 108 patients newly diagnosed with classical Hodgkin lymphoma (88% of patients had nodular sclerosis Hodgkin lymphoma; 82.5% were negative for Epstein-Barr virus) (281). High frequencies of abnormal MHC expression were also observed in another 233 patients with Epstein–Barr virus-negative classical Hodgkin lymphoma (83.2% for MHC class I and 46.8% for MHC class II) (282). Because classical Hodgkin lymphoma has a high ORR to PD-1 blockade, these data may suggest that non-T responses also play important roles in the effect of PD-1 blockade, which is supported by a study showing that after PD-1 blockade, genes implicated in cytolysis and natural killer cell function were upregulated in patients (283). In addition to natural killer cells whose antitumor function is MHC-independent, invariant natural killer T cells can be activated by signals from a lipid–CD1d complex (284), and alloreactive CD8 T cells demonstrated cytotoxicity effector function against MHC class I-deficient allogeneic cells in a TCR-independent manner (285). To enhance antigen recognition and T cell response, chimeric antigen receptor T cell therapies, bispecific T-cell engagers, oncolytic viruses, vaccination, and intratumoral IL-12 plasmid electroporation have been combined with PD-1/PD-L1 blockade (86, 286–290) but the clinical results are currently unavailable.

Second, because the absence of costimulation (“signal 2”) can result in T cell anergy (278), impaired costimulation could lead to ineffectiveness of PD-1/PD-L1 blockade. This is supported by recent studies showing that rescue of exhausted CD8^+^ T cells with PD-1 blockade requires CD28/B7 costimulation in a mouse model with chronic viral infection (291) and that response to PD-1 blockade requires the presence of both CD4^+^ and CD8^+^ T cells as well as CD28 and CD80/CD86 costimulation in a murine melanoma tumor model with low mutational load (165). However, an earlier study showed that PD-1 blockade *in vivo* leads to accelerated rejection of heart allografts only in the absence of CD28 costimulation, accompanied by expansion of alloreactive T cells and enhanced generation of effector T cells (292).

Although PD-1 is expressed only after T cell activation, which requires costimulation (9), it has been shown that PD-1 can be induced without CD28 costimulation (11); in fact, lack of costimulation leads to upregulation of PD-1 (16). In one study of patients with early-stage lung cancer, 10–80% of tumor-infiltrating CD8^+^ T cells were CD28^−^ (291). CD28 could be lost during aging, with repeated antigenic stimulation, and after exposure to some cytokines (293). Therefore, insufficient CD28 costimulation could be an important resistance mechanism for PD-1 blockade. Consistent with the high efficacy of PD-1 blockade in Hodgkin lymphoma, CD28 is strongly or moderately expressed on T cells surrounding CD80/CD86hi-expressing Reed-Sternberg cells (294–296). In contrast, chronic lymphocytic leukemia has no or low levels of CD80/CD86 expression on leukemia cells (297–299) with immunologic synapse formation defects (300) and is resistant to pembrolizumab in a clinical trial (224).

In addition to the CD28 pathway, the CD40–CD40L costimulatory pathway has been shown to be required for the ameliorative effects of anti-PD-L1 therapy and plays a critical role in rescue of exhausted CD8 T cells (301). Anti-CD40 agonists, which alone could effectively reverse cytotoxic T cell exhaustion by activating the mTORC1 pathway *in vivo*, significantly enhanced action of PD-1 antagonists in chronic infection *in vivo* (302). In addition, combining PD-1/PD-L1 blockade with costimulatory agonist antibodies to CD27 (164), CD137 (4-1BB) (303, 304), TLR3/7/9 (305–307) [TLR3 is also a safe vaccine adjuvant (308)], GITR (309), STING (310), or OX40 [the synergy to restore function of exhausted CD8^+^ T cells was only observed under helpless (no CD4+ T cell) condition (311)] have demonstrated enhanced antitumor effects in preclinical models. However, sequential (delayed anti-PD-1) but not concurrent anti-OX40 and anti-PD-1 treatment (combination) *in vivo* resulted in increased efficacy which required both CD4^+^ and CD8^+^ T cells (312).

Third, although anti-PD-1/PD-L1 antibodies block PD-1–PD-L1 interaction, they do not affect PD-1/L1 expression. Studies have demonstrated that expanded exhausted CD8^+^ T cells reactive to anti-PD-1/PD-L1 therapy *in vivo* retain high PD-1 expression (25); PD-1/PD-L1 blockade was shown to enhance IFN-γ and PD-L1 expression (42, 72) and increase tumor-infiltrating PD-1^+^ T cell frequencies (14). One preclinical study showed that the antitumor effect of anti-PD-1 therapy required the presence of PD-1^lo^CD8^+^ T cells before treatment and decreased frequencies of tumor-infiltrating PD-1^+^CD8^+^ T cells below a threshold after the anti-PD-1 therapy (24). However, clinical studies showed that PD-1^hi^ expression before treatment (263) or on treatment correlated with response to PD-1 blockade in melanoma patients (237).

High PD-1 expression as resistance mechanism is probably more relevant for anti-PD-L1 therapy, which only blocks PD-1–PD-L1 interaction by modulating cytosolic signaling pathways and does not reduce PD-1 expression. In a chronic LCMV infection model and a melanoma tumor model, anti-PD-L1 therapy did neither downregulate the *PDCD1* gene in treated T cells nor did reprogram the epigenetic landscape, including chromatin accessibility to Nr4a and NFAT transcription factors (14, 90).

Strategies to modulate the transcriptional (including epigenetic) and posttranscriptional regulation of PD-1/PD-L1 expression may lead to a more durable response in patients. The transcription factors and pathways positively regulating PD-1 expression include BLIMP-1 (although conflicting results were also reported) (313, 314), IFN-α–IRF9 (315), TGFβ–SMAD3 (316), NFATc1 (317), STAT3/4/NFATc1/CTCF (318), the Notch signaling pathway (319), FOXP1 (320), c-FOS (321), STAT1/2 (322), and NF-κB (323). In contrast, T-bet (324), trimethylation (37, 325, 326), and a chromatin organizer SATB1 (327) negatively regulate *PDCD1* expression. Chromatin accessibility to *PDCD1* enhancers (including the −23.8 kb enhancer) is important for PD-1 expression in exhausted T cells (328).

Fourth, insufficient antitumor activity may result from multiple T cell subtypes and subclones (including those with “fixed” T cell dysfunction) that are not responsive to PD-1/L1 blockade. Dysfunction of these T cell subclones may lead to tumor evolution of subclonal neoantigens, which were associated with primary and acquired resistance to checkpoint blockade in patients (250, 258). In a cancer model, “fixed” dysfunction of driver-antigen-specific T cells was associated with PD-1, TIM-3, LAG-3, and 2B4 expression (17). Although PD-1 has a uniquely critical role in immune suppression, co-expression of multiple immune checkpoint receptors on T cells resulted in greater T cell exhaustion (329).

Multiple blockade combinations have shown synergetic effects in releasing adaptive immune resistance in preclinical models (330), as well as combination strategies targeting the transcriptional program (17). Histone deacetylase inhibitors have been shown to increase expression of multiple T cell chemokine (paradoxically also PD-L1 expression) and enhance the response to PD-1 blockade *in vivo* (57, 331). EZH2 and DNMT1 inhibitors increased Th1-type chemokines and T-cell infiltration, and augmented the efficacy of PD-L1 blockade therapy *in vivo* (332). Simultaneous blockade of PD-1 and LAG-3 synergistically improved viral control and tumor eradication (329, 333, 334). Combined TIGIT and PD-1 blockade (335), or combined PD-1, TIM-3 (336), and BLTA blockade (337), increased the expansion and effector function of antigen-specific CD8^+^ T cells from melanoma patients *ex vivo*.

The combination of PD-1 blockade and CTLA-4 blockade, which has distinct immunologic effect and activates different T cell populations *in vivo* (283, 338), demonstrated greater antitumor effects than the use of either antibody alone (339, 340). Furthermore, clinical trials have demonstrated remarkable efficacy of combined nivolumab and ipilimumab therapy in melanoma (ORR: ~60%) (194, 341), although combined durvalumab (anti-PD-L1) and tremelimumab (anti-CTLA-4) in NSCLC was not successful in a recent phase 3 study (Table 3). Sequential use of nivolumab followed by ipilimumab or in reverse sequence did not reduce the toxicities resulting from concurrent (combination) therapy with nivolumab and ipilimumab, as found in a phase 2 study; nivolumab followed by ipilimumab showed higher response and survival rates but also higher toxicities compared with sequential use of ipilimumab followed by nivolumab, in which the synergistic effect was lost (342).

Fifth, the immunosuppressive tumor microenvironment may contribute to the in effectiveness of anti-PD-1/L1 treatment. Tregs, MDSCs, M2 macrophages, and their associated cytokines, chemokines, and other soluble factors are well-recognized inhibitory mechanisms orchestrated to suppress antitumor immunity (72). Depletion of tumor-infiltrating Tregs was shown to synergize with PD-1 blockade to eradicate established tumors *in vivo* (343). However, the clinical significance of Tregs was inconsistent in different studies, likely due to the differential function of Treg subsets (344). Moreover, as shown *in vivo*, the suppressive function of NRP1^+/+^ Tregs could be lost and converted to antitumor immunity in the presence of IFN-γ produced by HIF-1α^hi^ NRP1^−/−^ Tregs. This functional fragility signaled through the IFN-γ receptor was required for the effectiveness of PD-1 blockade *in vivo* (345).

Increased MDSCs have been shown to be associated with poor prognosis (346), whereas decrease in macrophages after anti-PD-1 therapy was associated with clinical response in melanoma patients (243). Combination of PD-1/PD-L1 blockade with tumor vaccines only partially restored the effector function of TILs stimulated by immunization and decreased Treg infiltration, but had little effect on the frequencies of MDSCs in the tumor lesions *in vivo* (19). Anti-PD-L1 blocking mAb augmented IFN-γ-mediated nitric oxide production by macrophages which inhibited CD4^+^ T cell proliferation; nitric oxide synthase inhibitor L-NMMA abrogated the inhibition and increased cytokine production (174). Indoleamine 2,3-dioxygenase (IDO) expression in tumor-associated macrophages and MDSCs induced by IFN-γ during CD8^+^ T cell response, can cause tryptophan deficiency and “metabolic checkpoint” in T cells (347, 348). Combining IDO inhibitors with anti-PD-1 therapy was shown to increase effector T-cell infiltration *in vivo* (349), and this combination has shown promising results in ongoing clinical trials (350). In addition, upregulation of *IL10* and macrophage/monocyte chemotactic genes was associated with resistance to anti-PD-1 therapy (259). Combination of PD-1 blockade with IL-10 neutralization *in vivo* resulted in reduced tumor burden and improved murine survival, accompanied by augmented antitumor function of T cells and decreased infiltration of MDSCs (351). However, recent clinical trials demonstrated that pegylated recombinant IL-10 combined with PD-1 blockade therapy enhanced the antitumor effect (352).

Moreover, a study showed that *in vivo* PD-1^−^ tumor-associated macrophages removed anti-PD-1 mAbs from the surface of PD-1^+^CD8^+^ T cells, mediated by the interaction between FcγII/III receptors and the anti-PD-1 Fc domain glycan (353). Therapeutic inhibition of FcγR interaction enhanced anti-PD-1 efficacy *in vivo*. Also, nivolumab was transferred from human CD8^+^ T cells to macrophages in an *in vitro* coculture system (353), although the IgG4 constant region sequences of nivolumab are designed to contain an S228P mutation to prevent antibody-dependent cell-mediated cytotoxicity and complement-dependent cytotoxicity (4). It is unknown whether pembrolizumab, which binds to PD-1 at a completely different region than does nivolumab (354), can also be transferred by this FcγR–mediated mechanism. Unlike anti-PD-1 mAbs, selective depletion of Tregs, dependent on activating Fcγ receptors expressed by macrophages, is essential for the activity of anti-CTLA-4 therapy *in vivo* (355, 356).

Sixth, systemic immunity is critical for tumor eradication and protection against new tumors; in the immune network, dendritic cell function and T cell infiltration play an important role (357). Gut dysbiosis (loss of microbial diversity) and antibiotic treatment were associated with shorter progression-free and/or overall survival in cancer patients receiving anti-PD-1 immunotherapy (271, 272). Conversely, improving the gut microbiome may lower the cancer-immune set point and circumvent resistance to PD-1 blockade (272). Peritumoral injection of LCMV alone or combined with PD-1 blockade has also been shown to induce immune surveillance and tumor regression *in vivo* (358).

## Concluding Remarks

Although the complexity of the PD-1/PD-L1 pathway has been revealed, our current understanding of the rejuvenation potential of T cells is only the tip of the iceberg. Accumulating evidence has demonstrated that PD-1 ligation suppresses the effector function of activated T cells; PD-L1 can directly cause tumor immune evasion; and anti-PD-1/PD-L1 mAbs that prevent PD-1–PD-L1 interaction can restore T-cell effector function. However, tumor PD-L1 expression through cell-intrinsic mechanisms may not have a significant role in driving immune suppression; PD-L1 and PD-L2 may also have costimulatory functions; and PD-1/PD-L1 blockade did not always elicit an effective antitumor response in preclinical studies. Moreover, although many anti-PD-1/PD-L1 clinical trials were remarkably successful which have revolutionized the treatment of cancer, some failed to reach the endpoint or resulted in an increased risk of death. In the setting of advanced cancers except Hodgkin lymphoma (likely also MSI-H tumors), only the minority of cancer patients had durable response to PD-1/PD-L1 blockade monotherapy, and some patients even had disease hyperprogression. Classical Hodgkin lymphoma, which does not have a high mutational burden or MHC class I expression, demonstrated a high response rate to PD-1 blockade therapy.

In addition to summarizing these paradoxical results in studies of PD-1/PD-L1 and PD-1/PD-L1 blockade, this review discussed a few open questions from mechanistic and clinical perspectives. As discussed, both PD-1 and PD-L1 are often (but not always) associated with T cell dysfunction; PD-1^+^ and PD-L1^+^ expression can also indicate T cell activation although PD-L1 and PD-1 may be expressed in different stages of immune response; markers to distinguish PD-1^+^ T cells with high functional avidity from exhausted PD-1^+^ T cells are unclear. Both PD-1 and PD-L1 can either dependently or independently drive immune suppression. Whether tumor or host factors dictate immunity remains to be determined. Mechanisms that are not completely understood also include those governing PD-1 expression, molecular pathways underlying PD-1/PD-L1 blockade, the difference in PD-1 signaling upon PD-L1 binding and upon anti-PD-1 mAb binding, metabolic crosstalk between tumor cells and T cells, and functional relationship (causal, consequential, or independent) between PD-1/PD-L1 expression and cell metabolism. Molecular delineation and critical node identification may also help clarify the inconsistent preclinical results of blocking PD-1 compared with blocking PD-L1.

It is unclear whether PD-1 blockade has different action (antagonist or agonist) in PD-L1^+^ and PD-L1^−^ patients. Also uncertain is whether this and other differences between PD-L1 and PD-1 (for example, the association of PD-L1 expression with earlier stage of immune activation, the more dynamic PD-1 expression, or other factors which are critical for immune response but differentially associated with PD-1 and PD-L1 expression) underlie the better predictive value of PD-L1 over PD-1 expression as a biomarker for clinical response. Tumor mutational burden has also emerged as a promising biomarker; however, our understanding of clonal mutations, T cell clonality, and neoantigen-reactive TIL clones responsive to PD-1/PD-L1 blockade may be still in its infancy. In addition, infiltration of immune cells, tumor immunogenicity, strength of TCR signaling and costimulation/co-inhibition, T cell differentiation stage and chromatin flexibility, immune cells and soluble factors in the tumor microenvironment, pharmacologic kinetics of antibodies, and systemic immunity may all affect the efficacy of PD-1 blockade. Future studies in the fast advancing field of immunotherapy may shed light on these intriguing questions, develop algorithms to accurately predict the blockade efficacy, and pave the way for a new era of combination immunotherapy.

## Author Contributions

ZX-M conceptualized and wrote the manuscript and created the figure. KY contributed to the conception and writing. MZ and JL revised the manuscript. All authors read and approved the final manuscript. The authors thank Erica A. Goodoff from the Department of Scientific Publications, MD Anderson Cancer Center, for her edition of the manuscript.

## Conflict of Interest Statement

KY receives research support from Roche Molecular System, Gilead Sciences Pharmaceutical, Seattle Genetics, Dai Sanyo Pharmaceutical, Adaptive Biotechnology, Incyte Pharmaceutical, and HTG Molecular Diagnostics.
